# Spatial connectivity in mosquito-borne disease models: a systematic review of methods and assumptions

**DOI:** 10.1098/rsif.2021.0096

**Published:** 2021-05-26

**Authors:** Sophie A. Lee, Christopher I. Jarvis, W. John Edmunds, Theodoros Economou, Rachel Lowe

**Affiliations:** ^1^ Centre for Mathematical Modelling of Infectious Diseases, London School of Hygiene & Tropical Medicine, London, UK; ^2^ Centre on Climate Change and Planetary Health, London School of Hygiene & Tropical Medicine, London, UK; ^3^ Department of Infectious Disease Epidemiology, London School of Hygiene & Tropical Medicine, London, UK; ^4^ Department of Mathematics, University of Exeter, Exeter, UK

**Keywords:** spatial analysis, infectious disease dynamics, epidemiology, vector-borne disease, machine learning

## Abstract

Spatial connectivity plays an important role in mosquito-borne disease transmission. Connectivity can arise for many reasons, including shared environments, vector ecology and human movement. This systematic review synthesizes the spatial methods used to model mosquito-borne diseases, their spatial connectivity assumptions and the data used to inform spatial model components. We identified 248 papers eligible for inclusion. Most used statistical models (84.2%), although mechanistic are increasingly used. We identified 17 spatial models which used one of four methods (spatial covariates, local regression, random effects/fields and movement matrices). Over 80% of studies assumed that connectivity was distance-based despite this approach ignoring distant connections and potentially oversimplifying the process of transmission. Studies were more likely to assume connectivity was driven by human movement if the disease was transmitted by an *Aedes* mosquito. Connectivity arising from human movement was more commonly assumed in studies using a mechanistic model, likely influenced by a lack of statistical models able to account for these connections. Although models have been increasing in complexity, it is important to select the most appropriate, parsimonious model available based on the research question, disease transmission process, the spatial scale and availability of data, and the way spatial connectivity is assumed to occur.

## 1. Introduction

The World Health Organization (WHO) estimates that over 80% of the world's population is now at risk of one or more vector-borne disease, accounting for 17% of the global burden of communicable diseases [1]. The past 50 years has seen an unprecedented emergence of mosquito-borne diseases, in particular dengue fever, chikungunya and Zika, linked to urbanization, globalization, international mobility and climate change [2,3]. Increased connectivity between geographical regions due to international air travel has led to these diseases invading previously naive populations where competent vectors exist, as seen in the introduction of chikungunya to Latin America and the Caribbean [4], and sporadic outbreaks of dengue fever in parts of Southern Europe [5]. Conversely, the global incidence of malaria has decreased over the past 20 years, with an increasing number of countries working towards eradication, although this trend has slowed in the past 5 years [6]. Spatial connectivity arising from human movement may pose a risk of re-introducing a pathogen into indigenous populations. Failure to account for this in modelling studies may negatively impact control and eradication campaigns [7].

The inclusion of space within infectious disease epidemiology is not a new phenomenon; however, the introduction of Geographical Information Systems, improvements in computational power, and availability of spatial data have made spatial modelling more accessible [8]. Despite this, Reiner *et al.* [9] found that spatial modelling methods were underrepresented in their review of mathematical models for mosquito-borne diseases, and spatial connectivity was not explored in the majority of studies. Tobler's first law of geography states that ‘everything is related to everything else, but near things are more related than distant things’ [10]. However, when studying mosquito-borne diseases, long-distance movement of hosts and vectors may create connections between distant regions. Connectivity between geographical areas and observations can arise for a number of reasons, for example, shared characteristics such as human behaviour, vector-control programmes, levels of immunity within communities and human and vector movement. Although these issues are common among diseases, their impact and the assumption about how connectivity arises may differ due to mosquito behaviours and different geographical settings.

Spatial connectivity is an important driver of mosquito-borne disease, but to our knowledge, there are no systematic reviews of spatial modelling techniques that include statistical, machine learning and mechanistic frameworks. These three approaches are used to address different objectives and require different types of information. Mechanistic models are less dependent on extensive training datasets than statistical or machine learning approaches and can be parameterized using previous experiments. However, this requires an in-depth understanding of the underlying disease process and incorrect parameterization could lead to invalid inference [11]. Mechanistic models are useful for studying (re-)emerging diseases, where few data exist, and comparing potential control strategies [12]. By contrast, machine learning models are able to make predictions about complex biological processes, without prior knowledge of the underlying process, using algorithms that learn from rich, complex data [13]. Statistical models are able to explore relationships between variables, test hypotheses about the underlying transmission process and make predictions about an outcome of interest where adequate data are available.

This systematic review aims to identify spatial models used to investigate the transmission of mosquito-borne disease to humans, the assumptions made about how spatial connectivity arises and the data used to inform the spatial models. We provide detailed explanations of these methods, their assumptions, how they were used, and discuss their advantages and disadvantages.

## 2. Methods

### 2.1. Search strategy

The PRISMA guidelines for systematic reviews and meta-analyses were followed for this review [14]. Five online bibliographic databases were searched: Ovid/Medline, Web of Science, Embase, Global Health and Scopus. The final search was completed on 14 December 2020. The search strategy included relevant keywords and Medical Subject Headings (MeSH) related to mosquito-borne diseases and the mosquito species that transmit them, mathematical models used to model infectious diseases and spatial connectivity. Full details of the search strategy are provided in table 1. Mosquito-borne diseases listed on WHO and European Centre for Disease Prevention and Control websites were considered: dengue fever, Zika, chikungunya, malaria, yellow fever, West Nile fever, Rift Valley fever, sindbis fever and Japanese encephalitis [15,16].

**Table 1.** Search terms used to search Medline, Embase, Global Health and Web of Science related to mosquito-borne diseases, modelling and spatial connectivity.

**mosquito-borne diseases**	**modelling**	**connectivity**
mosquito^a,b^ disease^b^	(math^b^ OR statistic^b^)^a^ model^b^	(spati^b^ OR cluster)^a^ analysis
chikungunya	(gravity OR radiation)^a^ model^b^	autocorrel^b^ OR neighb^b^ OR hierarch^b^ OR adjacen^b^ OR proximity OR network OR commut^b^ OR connect^b^
dengue	(spati^b^ OR Bayes^b^)^a^ model^b^	random^a^ effect^b^
‘Japanese encephalitis’	(ecolog^b^ OR environment^b^)^a^ model^b^	(BYM OR ‘Besag^b^ Yorke and Mollie’)^a^ model^b^
malaria	(dynamic OR stochastic OR determinist^b^ OR mechan^b^ OR compartment^b^)^a^ model^b^	‘conditional autoregress^b^’ OR CAR
(Rift Valley)^a^ (fever OR virus)	(regression OR general^b^)^a^ model^b^	human^a^ (mobility OR movement OR travel)
sindbis	(SIR OR SEIR)^a^ model^b^	spat^ba^ depend^b^
(‘West Nile’)^a^ (fever OR disease^b^ or virus)	patch^a^ model^b^	metapopulation
‘yellow fever’	(empirical OR correl^b^ OR movement)^a^ model^b^	spati^ba^ (structure OR matrix)
Zika		
Aedes		
Anopheles		
Culex		

^a^Proximity searching was used, search terms had to be within three words of each other. ADJ3 was used for Embase, Medline and Global Health, NEAR/3 was used for Web of Science.

^b^Denotes truncation. MeSH terms related to terms above were also searched.

Results from database searches were combined and stored using EndNote referencing software; duplicates were removed manually. The titles and abstracts were screened and irrelevant articles excluded. Two reviewers screened full texts independently and disagreements were resolved by consensus. After relevant papers were identified, their references were screened to identify other relevant studies.

### 2.2. Inclusion and exclusion criteria

The inclusion criteria are as follows: articles must be peer-reviewed, published in English and contain a spatial model that investigates the transmission of mosquito-borne disease to humans. Spatial models are defined as those that explicitly account for connections between geographical areas or observations. There were no geographical or publishing date restrictions applied. Articles were excluded if they only modelled transmission to vectors or non-human hosts as these were outside the scope of this review and may require different assumptions of connectivity. Theoretical modelling studies that were fitted using simulated data were excluded unless they were validated using real data. Conference and workshop proceedings were excluded, as were review articles.

### 2.3. Data analysis

The following variables were extracted from eligible papers: title, first author, year of publication, disease studied, country/region studied, the spatial scale of the data, spatial model used, the spatial method used to account for connectivity, connectivity assumptions and the data used to inform the spatial element of the model.

Spatial models were classified as either statistical, machine learning or mechanistic. Statistical models assume that the data are a realization of a pre-specified probability distribution. These probability distributions are defined by a set of parameters which are estimated from the data using estimation, inference and sampling techniques, such as maximum likelihood, Markov chain Monte-Carlo and bootstrapping. The association between an outcome of interest and a set of covariates is determined by how these affect the probability distribution of the outcome. Statistical models were also classified as either fixed effect, where all parameters are treated as fixed, non-random values or mixed effect, which contain both fixed parameters and random parameters that account for unobserved heterogeneity or clustering within the data. Machine learning methods use algorithms to learn patterns from observed data without the need to specify a data model prior to analysis. This makes them a useful alternative to mechanistic or statistical models where underlying biological processes are not known [13]. Mechanistic models, sometimes referred to as mathematical models, aim to replicate the process of disease transmission through a population across time based on a simplified mathematical formulation of the underlying disease mechanisms. These models often simulate the movement of individuals through infectious stages, or compartments, known as compartmental models [11]. Mechanistic models can be parameterized using a combination of data, when available, and results from previous studies. This makes them particularly useful for studying novel pathogens where there are few empirical data or when comparing potential control measures [12]. Spatial assumptions were compared between diseases and mosquito species.

Analysis of the data and visualizations were carried out using R [17]. Data extracted from the studies included in this systematic review and code used to create figures and tables are available from https://github.com/sophie-a-lee/mbd_connectivity_review and archived in a permanent repository [18]. This study is registered with PROSPERO, CRD42019135872.

## 3. Results

### 3.1. General characteristics

**Figure 1.**
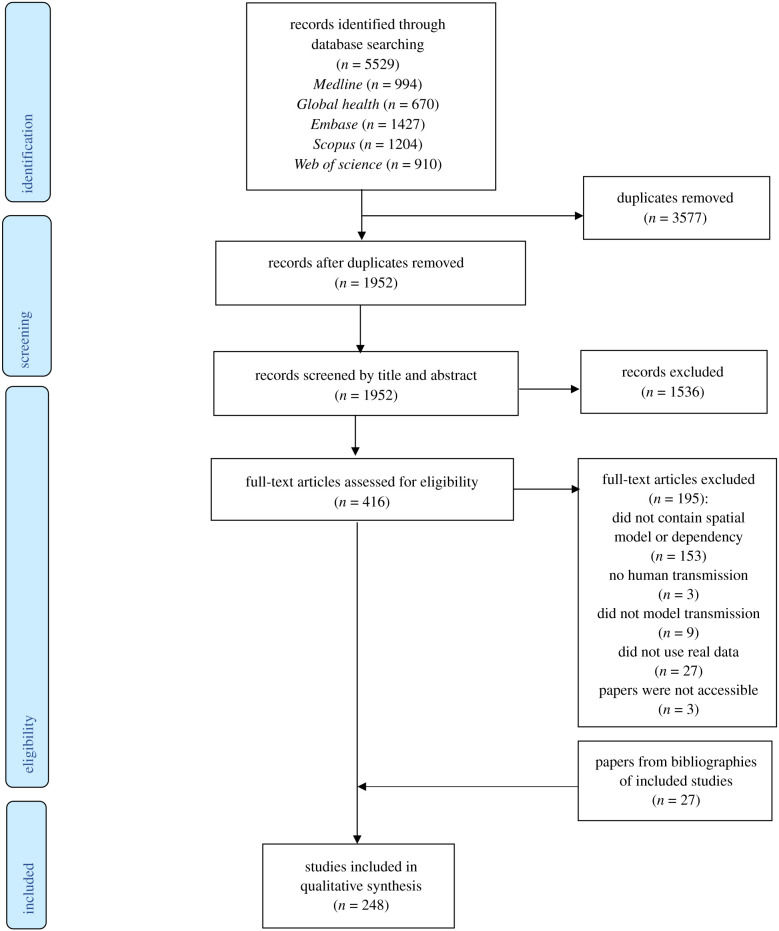
PRISMA flow diagram of the search and exclusion process.

We identified 248 studies published between 1999 and 2020 that were eligible for inclusion (figure 1). These studies used data from 164 countries across six continents (electronic supplementary material, figure S1). Almost half (*n* = 118, 47.6%) of the studies modelled malaria transmission, 99 (39.9%) modelled dengue fever (including two modelling dengue haemorrhagic fever, two which also modelled Zika, one that also modelled chikungunya and one that modelled dengue, chikungunya and Zika), 11 (4.4%) modelled just Zika and five (2%) just chikungunya, one modelled both. Seven (2.8%) modelled West Nile fever, five (2%) Japanese encephalitis, 1 (0.4%) Rift Valley fever and one (0.4%) yellow fever. No spatial modelling studies were identified for sindbis fever. The number of spatial modelling studies published has increased over time, with an average of one study published per year in 1999–2005, 5.8 per year 2006–2010, 14.2 per year 2011–2015 and 28.2 per year 2016–2020. The diversity of diseases studied using spatial modelling has also increased; until 2005, only malaria studies were identified whereas there have been six different diseases studied using these methods published in 2020 (electronic supplementary material, figure S2). Most studies (*n* = 218, 87.9%) used aggregated data to fit models, most often aggregated to administrative district- or country-level (*n* = 169, 68.1%) or clusters based on surveys or shared characteristics (*n* = 25, 10.1%). The remaining papers either separated their study area into a grid and aggregated data to these patches (*n* = 24, 9.7%) or fit data to individuals (*n* = 30, 12.1%). A full summary of data extracted from studies by disease is given in electronic supplementary material, table S1.

### 3.2. Spatial modelling methods

**Figure 2.**
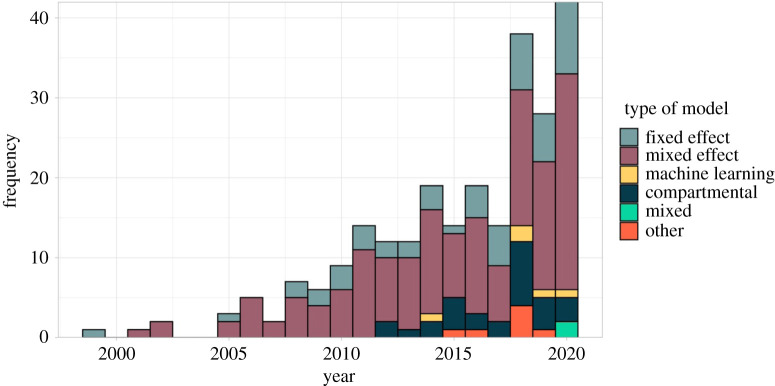
Number of spatial modelling studies published per year by model type. Statistical models were classified as a fixed effect if parameters were treated as fixed, non-random values or mixed effect if they also included random parameters to account for unobserved heterogeneity or clustering (also known as hierarchical or multilevel models). Machine learning models used algorithms to learn patterns from the data. Compartmental models were mechanistic models that simulated the movement of hosts and/or vectors through disease compartments. Models classified as 'other' did not fall into any of these categories, this included mechanistic models that did not explicitly model movement through compartments, or bespoke statistical models.

Most (*n* = 209, 84.2%) studies used a statistical modelling framework, in particular mixed effect models (*n* = 155, 62.5%). The first mechanistic model included in this review was published in 2012; mechanistic models are becoming more common with over half of those studies published since 2018 (figure 2). Newly emerging diseases (Zika and chikungunya) were more often modelled using mechanistic models rather than statistical, which were more commonly used for established diseases (e.g. malaria and dengue) (electronic supplementary material, table S1). There were two studies published in 2020 that used a combination of methods: one compared a mechanistic and machine learning approach to predicting dengue transmission [19], another used both a machine learning and statistical approaches to explore the relationship between risk factors and dengue outbreaks [20].

We identified 17 distinct models that incorporated spatial connectivity into their framework: nine statistical, four machine learning and four mechanistic models. Full descriptions of the 17 models identified in this review, including model structure, the method and data used to account for spatial connectivity, and a discussion about the advantages and disadvantages of each model are given in electronic supplementary material, technical appendix. Some models were specifically designed for spatial analysis, whereas others have been adapted or extended to incorporate this connectivity. This section gives an overview of the methods used to account for spatial connectivity for each type of model. Details and best practices are summarized in table 2.

**Table 2.** The advantages, disadvantages and uses of spatial modelling methods.

model type	spatial method	description	advantages	disadvantages	application
statistical or machine learning	spatial covariate	inclusion of a covariate that aims to describe spatial connectivity within a regression model. For example, incidence of surrounding regions, distance between observations, or number of people moving between regions. The covariate is treated as a fixed effect and included into a model as any other covariate	compatible with all statistical or machine learning methods relatively quick and simple to fit allows human and vector movement to be included interpretation of coefficients is often simpler than other methods allows models to 'borrow strength' from connected regions, improving precision	models assume that the relationship between the outcome and spatial covariates is stationary and isotropic inclusion of a large number of spatial covariates increases risk of overfitting and multicollinearity within a model user must specify which regions/observations are connected prior to model fitting which does not allow other connections to be explored	exploratory tool for statistical or machine learning studies carried out on a small scale where few spatial connections are expected. Statistical or machine learning modelling studies where spatial connectivity is assumed to arise from human movement
statistical	local regression models	local regression models are fitted to each region using data from nearby regions, weighted by distance. Also known as GWR. Coefficients are calculated separately for each regression model	relatively simple to carry out and interpret useful exploratory tool to understand how the relationship between covariates and the outcome differ across space does not assume these relationships are stationary	does not provide a global model to make interpretations about a region as a whole only allows distance-based spatial connectivity to be included	exploratory tool to generate hypotheses about how relationships differ across space. Cannot be used to make inferences about regions as a whole. Only appropriate when studying areal data
statistical	random effects and fields	random effects or fields with a spatially structured covariance function are included in a regression model to account for additional correlation or heterogeneity arising from spatial connectivity. Users must choose an appropriate spatial structure before fitting the model, usually assuming that regions are connected if and only if they are adjacent (areal data) or that connections decay exponentially as the distance between them increases (individual-level data)	relatively easy to obtain connectivity data (if using structure based on adjacency or distance) does not assume stationarity in the model allows connections between a large number of observations without issues of overfitting associated with other statistical methods increasing number of methods and software developed to make model-fitting process simpler	more complex to fit and interpret models than other statistical models random effects require an appropriate spatial structure defined before model is fitted structures identified in this review only allow models to account for connectivity between neighbours or close regions, other connectivity has not been explored	statistical models where spatial connectivity is expected to exist between nearby regions. Can be carried out in small- or large-scale studies. Recommended for established diseases rather than a newly emerging setting as requires large amounts of data for precise estimates
machine learning	movement matrix	movement matrices reflecting the movement of humans around a network used to weight connections between hidden layers of a neural network	allows complex, dynamic connectivity structures to be explored allows human movement to be included in a machine learning framework	requires human movement data (or a representative proxy) to create which can be difficult to obtain inclusion of the matrix in the hidden layer of neural networks means the impact of this movement is difficult to observe computationally intensive	inclusion in a neural network where human mobility is known to drive transmission. Studies that require accurate predictions based on a large amount of data but quantifying this process is not the focus
mechanistic	spatial parameter	spatial parameters are included in mechanistic model equations, either to take account for a spatial process or to update populations within each disease compartment of the model. Examples include diffusion parameters allowing hosts and vectors to move across a region or mosquito abundance that borrows information from connected regions	models can be fitted with few data and used to make causal inferences parameters can borrow information from other regions about processes to take account of shared characteristics less computationally intensive to fit than other mechanistic approaches can be used within any mechanistic model	requires knowledge and information regarding the underlying process of transmission parameters assume that the impact of spatial coefficients on transmission is stationary within a compartmental model making them inappropriate on a large scale	models aiming to make causal inferences about the underlying process of transmission. Able to fit models where few data are available making it useful for newly emerging diseases or areas with low transmission. More appropriate in small-scale studies where stationarity can be assumed
mechanistic	movement matrix	movement matrices that reflect the movement of hosts and/or vectors around a network are included within a mechanistic model. These allow interaction between hosts and vectors in different locations and update the population at each node of the network	allows complex, dynamic connectivity structures to be explored results can be extrapolated beyond the data used to fit them and causal inferences can be made provides a more 'realistic' reflection of human and vector behaviour models can be fit with relatively few data	adequate movement data are difficult to obtain the complex nature of these models means computation can be difficult and time consuming inferences can only be made about the setting the model is parameterized to reflect requires the population being studied to be split into nodes in networks	models taking account of human and/or vector movement or other complex connectivity structures. Able to fit models where few data exist as well as large amounts, useful for newly emerging diseases. Able to study the process of transmission or causal structures. Works well with agent-based or metapopulation mechanistic models where the population is described using a network

#### 3.2.1. Statistical models

All statistical models identified within this review were extensions of generalized linear or additive models (GLM/GAM). These models assume that all observations are independent after adjusting for the covariates, which is not always appropriate when considering spatial data. Although there were nine distinct statistical models, all of them used one of three methods to account for spatial connectivity: inclusion of spatial covariates as fixed effects, localized regression models or the inclusion of a spatially structured random effect or random field.

##### 3.2.1.1. Spatial covariates

Of the 209 papers using statistical models, 25 (12%) included spatial covariates to account for spatial connectivity in the data. Spatial covariates are entered into the model in the same way as nonspatial covariates, but aim to account for connectivity within the model. Spatial covariates included the observed incidence in connected regions [21–30], the number of people moving between regions [20,31–35], the distance between regions [31,35–37], coordinates of the centroid of a region [38–40], the number of time spent commuting between regions [41] and spatial eigenvectors created using spatial filtering [42–44]. Spatial filtering creates spatial covariates by decomposing Moran's I (a measure of spatial correlation) into an eigenvector per region/observation [45]. Two studies applied a smoothing function to the spatial covariates within a GAM, allowing for a nonlinear relationship between the outcome and measure of connectivity [24,37]. Another study included spatial kernels, exponentially decaying correlation functions of the distance between cases' home and work addresses, estimated from public transport journeys, as spatial covariates when estimating the probability of cases being linked [46].

Spatial covariates are compatible with all statistical models identified in this review. If adequate data are available, this is a simple and efficient way to include connectivity information into a statistical model. Using information from connected regions also allows the model to ‘borrow strength’ from other parts of the data to increase the precision of estimates. Spatial covariates were the only method that allowed human movement data to be included in statistical models identified in this review; all other methods relied on a function of distance. However, the inclusion of a large number of spatial covariates risks overfitting the model to the data, meaning the model reflects the sample data too closely and is unable to make prediction or inferences about the wider population, or introducing multicollinearity. Most spatial covariates require ‘connectivity’ to be defined prior to model fitting, introducing a subjective element into the model and potentially oversimplifying the spatial structure. For example, models that included incidence from connected regions defined these as regions that share borders; this ignores potential dependency between distant regions which could still invalidate the independence assumption. The inclusion of spatial covariates as fixed effects assumes that the relationship between them and the outcome is stationary (the same across the whole spatial area) and linear which may not be appropriate across large areas.

##### 3.2.1.2. Local regression models

**Figure 3.**
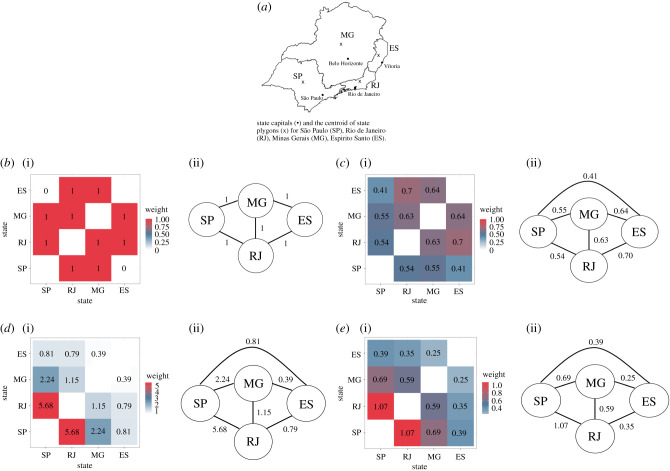
Comparison of spatial connectivity using different data sources and assumptions. The level of connectivity between regions represented in models can differ substantially depending on the assumptions made about how connectivity arises, and the data used to weight connections. The heat plots and connectivity matrices show the strength of connectivity between states in Southeast Brazil (*a*), represented by nodes in the matrices, using assumptions and methods identified in this review. Numbers within the heat plot and along edges of the connectivity matrix represent the weight of connections. These techniques were used to weight observations in GWR models, to structure random effects and random fields, or to weight movement matrices in neural networks, metapopulation models, and agent-based models. (*b*) Neighbourhood based: assumes states are connected if and only if they share a border. Application: to structure random effects in a CAR model. (*c*) Distance-based: assumes connectivity between states decays exponentially as distance between centroids (denoted x on the map) increases, where *weight = exp(dij /1000)* and *dij* is the distance between states *i* and *j*. Application: used to weight observations from neighbouring regions in a GWR model. (*d*) Human movement data: assumes connectivity between states arises due to human movement. In this case, based on the number of air travel passengers moving between capital cities of each state. Application: to weight hidden layers within a neural network. (*e*) Movement model: assumes connectivity between states arises due to human movement, estimated using a movement model (in this case, a gravity model). Application: used to weight movement between nodes in a metapopulation model.

Twenty papers used a geographically weighted regression (GWR) model [47–65] which fits local regression models to each observation or region rather than a single global model [66]. Each local model has different coefficients, estimated using information from connected observations that are weighted by a function of distance, such as the one shown in figure 3*c*. As with spatial covariates, GWR is a fairly simple and efficient method to account for connectivity and a useful exploratory tool to investigate how relationships differ across space. Estimating a different coefficient for each model overcomes the issue of stationarity which is present when using spatial covariates. GWR is not suitable for making inferences or predictions about the study area as a whole.

##### 3.2.1.3. Spatially structured random effects and random fields

The final, and most common, method used to account for spatial connectivity in statistical methods was the inclusion of a spatially structured random effect or random field. Fixed effect statistical models assume that there is a true parameter value and that the only variation within the data, after accounting for covariates, is sampling error. Random effects and random fields explicitly allow additional spatial variation and/or correlation in the data to be incorporated directly into the model structure. The structure of the random effects or random fields must be specified prior to model fitting and should be informed by the spatial connectivity assumption. Most models identified in this review used a Gaussian process which assumes the spatial process at fixed locations follows a multivariate normal distribution, with a mean of 0 and a covariance structure based on distance or, when dealing with areal data, adjacency.

We identified 150 studies (150/209, 71.8%) that used a spatially structured random effect within their statistical model, 95 assumed a Markov random field structure based on adjacency [29,40,42,64,67–156] and 57 used a distance-based structure [141,157–212] (one used both [141]). A commonly used Markov random field is known as the conditional autoregressive (CAR) model, which assumes that regions are connected if and only if they are neighbours [213], i.e. regions that share a border or, in one case, regions within a fixed distance [140]. The weighting matrix used to formulate this Markov random field is shown in figure 3*b*. Distance-based approaches identified in this review used the Matérn correlation function [214] to define the random effect covariance. This assumes that connectivity between points decays exponentially as the distance between them increases, as shown in figure 3*c*. There were 15 studies that included a spatially structured random field, a bi-dimensional smooth function in space over the coordinates of observations or the centroid of a region [40,215–227]. Bi-dimensional smooth functions are a type of Gaussian process, with a covariance structure defined by the distance between observations, for which connectivity is expected to decrease exponentially as distance increases [228] (figure 3*c*). One spatial model included a random field, estimated using a Markov random field [229], similarly to the CAR models above, assuming connectivity exists between neighbouring regions [228]. One study used an alternative way of accounting for residual spatial autocorrelation by fitting a separate regression model to the error terms of a non-spatial model. The observed outcomes from previous time points were included in the residual model as covariates. This model was fitted using an iterative process and was referred to as a vectorial autoregressive model [230]. Further details are given in electronic supplementary material, technical appendix.

Although random effects and random fields are more computationally intensive than the other statistical approaches, there are a number of statistical methods and programs built to fit these types of models which aim to overcome computational issues [228,231,232]. These models are able to account for dependency between a large number of regions or observations without overfitting or introducing multicollinearity that causes issues when using spatial covariates. The structure of random effects and random fields must be determined before the model-fitting process, potentially introducing subjectivity into the model-fitting process, although they can be visualized which can help generate hypotheses and identify additional factors that may not have been accounted for within the original model. Within this review, we only identified two spatial structures that were used within these models: distance based and neighbourhood based. These structures are adequate if spatial connectivity exists between close observations but we did not identify structures that would allow for other assumptions, such as long-distance movement of hosts and vectors, to be incorporated into a statistical model.

#### 3.2.2. Machine learning methods

We identified two methods that were used to account for spatial connectivity within machine learning models: the inclusion of spatial covariates, and the development of movement matrices that aim to replicate human movement behaviour.

##### 3.2.2.1. Spatial covariates

Five papers included spatial covariates as inputs for their machine learning algorithms. These spatial covariates included cases from neighbouring regions [233–235], the number of people travelling between regions based on air travel [234], public transportation networks [20] or a gravity model that aimed to replicate human commuting behaviour [236], and the distance between countries [236]. The inclusion of spatial covariates as inputs is compatible with all machine learning models and, if the data are available, does not require any additional computation.

##### 3.2.2.2. Movement matrices

We identified two papers that constructed a matrix reflecting the movement of people between districts using public transportation data [19,237]. Both papers used this matrix, similar to the one shown in figure 3*d*, to weight layers within a neural network model, allowing the algorithm to predict the number of dengue cases across the study area while accounting for connectivity arising from human mobility. Although both studies used public transportation information to create their matrices, they could be constructed using movement models that aim to replicate human commuting behaviour, such as gravity or radiation models [238] (figure 3*e*), or other proxies such as distance-based functions where data are not available (figure 3*c*).

#### 3.2.3. Mechanistic models

There were two methods used to account for spatial connectivity in mechanistic models identified by this review: movement matrices and spatial parameters.

##### 3.2.3.1. Movement matrices

There were 21 studies (21/34, 61.8%) included in the review that used a movement matrix within a mechanistic model to account for spatial connectivity [19,32,239–257]; all these studies assumed that connectivity arose from either host or vector movement. These models treated subgroups of the host and/or vector populations as nodes in a network with values of the matrix reflecting movement between those nodes. Examples of these matrices constructed using different assumptions and data are given in figure 3. Matrices were constructed using human movement data from Twitter [32,251,256], air travel [239,249,250] or public transportation [19], using movement models that aimed to replicate human commuting behaviour [32,241,243,244,246,248,254,255,257], distance [242] or using a fixed value based on the type of neighbourhood [252,253]. Two studies estimated people's home and work addresses using mobile phone data and simulated movement between those [245,247], and two simulated the short flight distance of mosquitoes by allowing movement into neighbouring cells [240,245].

##### 3.2.3.2. Spatial parameters

Thirteen studies (13/34, 38.2%) included spatial parameters within the model equations that aimed to account for connectivity [67,258–269]. Unlike movement matrices, these were directly incorporated into the model equations to update the population within a given compartment, or as a proxy for another process. Spatial parameters included the force of infection calculated using a distance-based kernel [259,260] and mosquito abundance estimated using a GAM containing a spatial random field [258]. Some models updated the population within compartments based on spatial parameters, either using a fixed-distance dispersion value [264–266], or calculating the proportion leaving regions using mobile phone records [263], air travel [262] or movement models [262,269]. One study used a mechanistic model but estimated the number of infected people using a CAR model [67].

### 3.3. Spatial connectivity assumptions

We collected details on the assumptions that authors made about how spatial connectivity arises within the data, regardless of the model type or method used. Although the exact assumptions differed between studies, all could be grouped into one or more of the following categories:

1. distance based,
2. human movement,
3. vector movement.

This section presents the advantages, disadvantages and methods used to implement these assumptions. A summary of these points with guidance on their ideal uses are provided in table 3.

**Table 3.** The advantages, disadvantages and application of connectivity assumptions.

connectivity assumption	advantages	disadvantages	application
distance based	easy to obtain data can be incorporated into all types of model can be used as a proxy for shared characteristics that cannot be observed	oversimplifies process of transmission misses connectivity between distant regions difficult to define how ‘close’ regions should be to be considered connected	small-scale studies where unobservable processes, such as shared behaviours, create spatial connectivity. Not appropriate where long-distance connections are expected to exist due to travel. Basis of most statistical approaches identified in this review, e.g. GWR and mixed effect models
human movement	shown to be an important part of disease transmission for mosquito-borne diseases can account for connectivity between distant observations as well as close	difficult to quantify and obtain data, often requiring a proxy such as distance to be used data often have a number of biases may not be necessary for malaria studies in small-scale studies of endemic areas	*Aedes* or *Culex*-borne diseases in endemic settings where commuting leads to increased exposure, studies in areas that are disease-naive or nearing elimination at risk of (re-)introduction from long-distance movement such as immigration. More popular in mechanistic approaches such as metapopulation or agent-based models that allow complex movement matrices to incorporated. Only spatial covariates were able to reflect this connectivity in statistical methods
vector movement	an important part of the disease transmission process for all mosquito-borne diseases	difficult or impossible to obtain data due to the short flight distances of most mosquitoes, would not be necessary if considering a large area or a short-term study	small-scale studies or long-term forecasts, particularly malaria studies where transmission generally occurs at night. Due to a lack of data, a proxy must be used such as distance based on known flight distances of mosquitoes. May be included to account for differences in exposure levels across space

#### 3.3.1. Distance based

There were 200 (200/248, 80.6%) studies that assumed connectivity existed between observations or regions if and only if they were close. Although this was by far the most common assumption observed in this review, it was not explicitly stated in many of the studies. Twenty-two studies stated that they used a distance-based assumption as close regions were more likely to share characteristics such as climate systems, protective behaviours (e.g. bed net use), socioeconomic and demographic factors, vector ecology and land use type.

The majority of studies making a distance-based assumption of connectivity used a statistical model, only five studies used a mechanistic model and three used machine learning. The most common method for including distance-based connectivity within a model was the inclusion of a random effect or random field with a covariance structure defined by distance or neighbours (*n* = 162). Other methods included using spatial covariates (*n* = 16), such as the incidence rate in neighbouring regions or distance between observations, and local regression models fitted using data from nearby regions, weighted by distance (*n* = 20).

One of the main advantages of making a distance-based assumption of connectivity is that measures of connectivity (either distance or contiguity) are easy to obtain from geographical data. Contiguity is usually defined with chess analogies: rook contiguity defines neighbours as those sharing a common edge or border, whereas queen contiguity also includes regions sharing a common vertex. Another advantage of using one of these approaches is that there are a number of well-established models (particularly in statistical analysis) that were designed or adapted to incorporate this information, such as GWR and CAR models.

The main drawback of assuming connectivity is solely based on distance is that it may oversimplify the process, particularly for mosquito-borne diseases which require interaction between a susceptible host and an infectious vector. One of the most common models based on the assumption that connectivity exists between neighbouring regions, the Besag, Yorke and Mollié model (one example of a CAR model), states that these assumptions are reasonable if the disease is non-contagious and rare, which is not the case for mosquito-borne diseases [273]. Although regions are more likely to share characteristics with close regions, it is hard to define where this ‘closeness’ ends and how similar places should be before they are considered connected. Most studies assumed that characteristics were shared between neighbours or within a set distance; however, applying the same rule for all shared characteristics may miss some heterogeneity or exaggerate connectivity.

#### 3.3.2. Human movement

**Figure 4.**
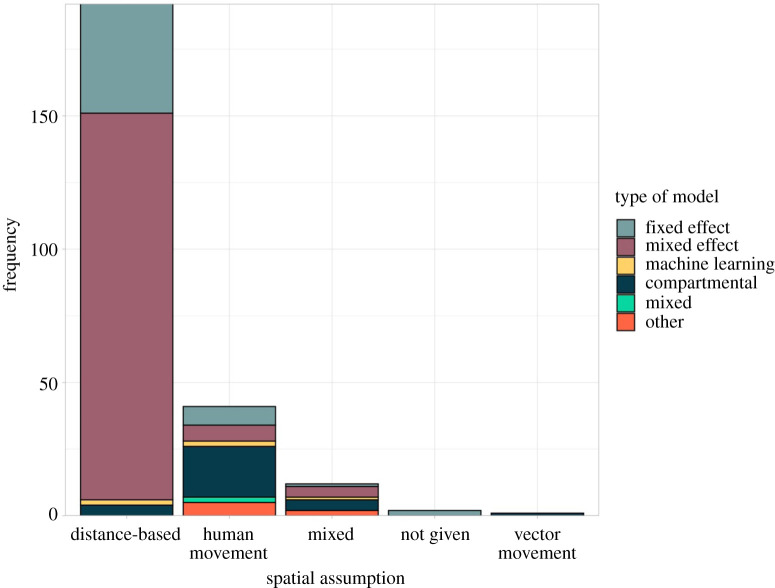
Connectivity assumption by model type. The number of spatial modelling studies that assumed connectivity is based on distance, human movement or vector movement (bars) separated by model type. The vast majority of statistical models (fixed and mixed effect models) assumed that connectivity was based on distance, whereas compartmental models were more likely to assume human movement drives connectivity.

We identified 50 studies that assumed spatial connectivity was related to human movement; most used mechanistic models (*n* = 28, figure 4) which are able to include complex mobility matrices (see metapopulation and agent-based models in electronic supplementary material, technical appendix, and figure 3 for more details). Other methods used to account for human movement within models included spatial covariates based on the number of people moving between regions, random effects which assumed people were more likely to travel to neighbouring regions, and a bespoke statistical model which simulated home and work addresses based on public transport journeys [46].

**Figure 5.**
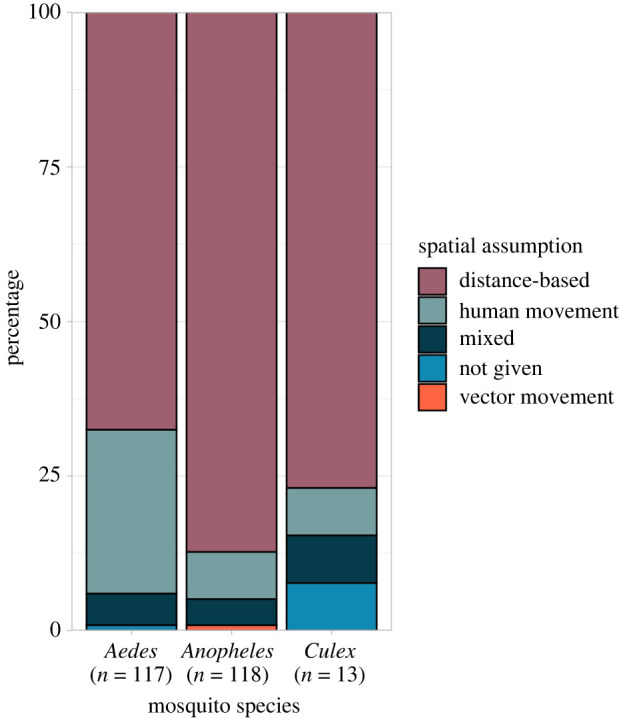
Connectivity assumptions by mosquito species. The percentage of studies modelling a disease transmitted by each mosquito species that assumed spatial connectivity is related to the distance between regions or observations (using a distance-based function or a neighbourhood structure), human movement or vector movement. Dengue fever, chikungunya, yellow fever and Zika were transmitted by mosquitoes of the *Aedes* genus; malaria was transmitted by mosquitoes of the *Anopheles* genus, and Japanese encephalitis, Rift Valley fever and West Nile fever were transmitted by mosquitoes of the *Culex* genus.

Studies were more likely to assume spatial connectivity arose through human mobility if the disease was transmitted by a mosquito of the *Aedes* genus (figure 5); this included dengue fever, chikungunya, yellow fever and Zika. *Aedes* mosquitoes are most active during the day, meaning interaction between host and vector is influenced by commuting behaviour [274], whereas *Anopheles* mosquitoes are night-biters and are more likely associated with vector movement or migration [275,276]. Less than half (*n* = 22) of the studies in this group used human mobility data to inform the spatial component of the model. Human mobility datasets included mobile phone GPS data, geo-located tweets, air travel information, public transportation networks and surveys. Other studies used a proxy such as distance or movement models, which replicate human commuting behaviours. The most common movement models were the gravity and radiation models. Both models assume that the movement of people is related to the population at each location and the distance between them; the radiation model also takes account of the population between locations under the assumption that people are less likely to commute to distant places when opportunities exist closer to home [238].

Unlike distance-based methods, the human mobility assumption allows for long-distance connections which may be important to the disease process, particularly in the region at risk of (re-)introduction of disease from imported cases. Prior studies have identified the importance of human mobility in the transmission of mosquito-borne diseases and found that failure to adequately account for this can lead to biased or invalid inferences [7,32,247,263,272,274,277]. However, human movement data can be difficult to obtain and may not be representative of all demographic and socioeconomic groups [272].

#### 3.3.3. Vector movement

We identified 10 studies that explicitly stated they assumed spatial connectivity arose from vector movement; all these studies used a fixed distance or adjacency as a proxy for vector movement as adequate movement data was not available. One model included wind speed to account for vector movement as this extended the potential flight distance of mosquitoes, another weighted vector movement to adjacent tiles making this more likely if adjacent tiles contained humans or breeding grounds. There was only one study in this review that assumed all connectivity arose from vector movement, all others included other assumptions.

## 4. Discussion

This review provides the first comprehensive overview of spatial models, of any type, used to investigate the transmission of mosquito-borne pathogens, and the connectivity assumptions that underpin them. The last 10 years have seen a rapid increase in the number of spatial modelling studies of mosquito-borne diseases and the variety of approaches used. We identified 17 distinct spatial models that were used to explore the transmission of mosquito-borne pathogens to humans. These were classified as either statistical, machine learning or mechanistic; the choice of model should depend on the aim of the study, the type of data available and the information required from the modelling output. Statistical models are able to explore relationships between variables when sufficient data are available and can be used to make predictions or inferences about an outcome of interest. Unlike mechanistic models, they do not require an in-depth knowledge of the underlying biological process of the disease, although this can be used to improve the model. However, statistical models require a large amount of data to provide precise estimates, making them more suited to well-established diseases. They are able to make predictions within the scope of the data used to fit them but are not recommended for causal investigations or extrapolation well beyond the data. Mechanistic models are more able to make causal inferences as they model the disease transmission process rather than the data itself; however, they are only able to do this within the specific setting for which they have been parameterized. Parameters can be taken from previous experiments where data are not available, making them particularly useful in settings where data are sparse or for newly (re-)emerging diseases. An example of this can be found in Zhang *et al*. [239] where parameters were 'borrowed' from other settings. Care should be taken when parameterizing mechanistic models in this way as processes may differ in ways that are not apparent at the model-fitting stage. By contrast, machine learning methods require a large amount of data but use flexible algorithms that allow them to learn patterns from rich, complex data. Although machine learning can be used to make inferences about data, most algorithms focus on making the most accurate predictions possible from available data rather than understanding underlying associations [270]. As with statistical models, they are inappropriate where there is a lack of data and are not recommended for making predictions or causal inferences well outside the range of data used to fit them [271].

Connectivity assumptions differed between mosquito species, indicating that authors consider mosquito behaviour and biting patterns when deciding which spatial model and assumptions are most appropriate. For example, dengue fever is transmitted by day-biting *Aedes* mosquitoes and is influenced by local movement or commuting [274], whereas *Anopheles*-borne malaria is transmitted by vectors most active between dusk and dawn so is influenced by proximity to vector breeding grounds and bed net use [275,276]. *Anopheles*-borne pathogens were more likely to be modelled assuming connectivity was driven by distance, potentially a proxy for vector movement because of the short flight span of vectors. *Aedes-* and *Culex-*borne pathogens were more likely modelled assuming human movement or proximity drives connectivity as this accounts for people commuting or moving to nearby regions/cities (figure 3). An alternative explanation could be that *Aedes-*borne emerging diseases (e.g. chikungunya and Zika) were more likely to be modelled using a mechanistic framework, allowing for the inclusion of complex movement matrices. The majority of statistical models within this review included a random effect to account for spatial connectivity, all of which used either a distance- or neighbourhood-based covariance structure. There were no random effect model structures that explicitly adjusted for connectivity arising from human movement.

Many studies included in this review did not explicitly state the assumptions they made about how connectivity arises. Often, assumptions had to be deduced from the data and spatial methods used in the studies. Although the vast majority of studies appeared to assume that regions were connected to neighbours or based on the distance between them, it is possible some used this as a proxy for another assumption, such as shared characteristics or human movement, where data were not available. Prior studies have discussed the difficulty of quantifying human behaviour when modelling infectious diseases [272]. Where mobility data are not available, movement models that aim to replicate commuting patterns, such as gravity and radiation models, were found to give similar results when modelling the spread of dengue fever compared to actual human movement data from geo-located Tweets [278]. These may help to avoid some of the issues surrounding privacy and bias when using a mobile phone or social media data to inform models, and where certain sections of the population, such as children and older adults, may be under represented. Some studies have suggested that radiation models are more accurate at representing commuting networks than mobile phone GPS data when compared to official census surveys in central locations [279].

This review provides a synthesis of the modelling approaches and spatial connectivity assumptions used to research mosquito-borne disease transmission to humans, but does not comment on the quality of these approaches. It is important to remember that more complex methods are not necessarily better and care should be taken to identify the most parsimonious method to address a studies' aim. Choice of the model should depend on the research question, the disease studied, the spatial scale and availability of the data and the way in which spatial connectivity is assumed to occur.

## Data accessibility

Data extracted from the studies included in this systematic review are available from https://github.com/sophie-a-lee/mbd_connectivity_review and archived in a permanent repository on Zenodo (http://doi.org/10.5281/zenodo.4706866) [280].

## References

[1] World Health Organization. 2017 UNICEF. Global vector control response 2017–2030.

[2] Wilder-Smith A, Gubler DJ, Weaver SC, Monath TP, Heymann DL, Scott TW. 2017 Epidemic arboviral diseases: priorities for research and public health. Lancet Infect. Dis. **17**, e101-e106. (10.1016/S1473-3099(16)30518-7) 28011234

[3] Paixão ES, Teixeira MG, Rodrigues LC. 2018 Zika, chikungunya and dengue: the causes and threats of new and re-emerging arboviral diseases. BMJ Glob. Health. **3**(Suppl. 1), e000530. (10.1136/bmjgh-2017-000530) PMC5759716 29435366

[4] Khan K et al. 2014 Assessing the origin of and potential for international spread of chikungunya virus from the Caribbean. PLoS Curr. **6**. (10.1371/currents.outbreaks.2134a0a7bf37fd8d388181539fea2da5) PMC4055609 24944846

[5] Schaffner F, Mathis A. 2014 Dengue and dengue vectors in the WHO European region: past, present, and scenarios for the future. Lancet Infect. Dis. **14**, 1271-1280. (10.1016/S1473-3099(14)70834-5) 25172160

[6] World Health Organization. 2020 World malaria report 2020: 20 years of global progress and challenges.

[7] Prothero RM. 1977 Disease and mobility: a neglected factor in epidemiology. Int. J. Epidemiol. **6**, 259-267. (10.1093/ije/6.3.259) 591173

[8] Auchincloss AH, Gebreab SY, Mair C, Diez Roux AV. 2012 A review of spatial methods in epidemiology, 2000–2010. Annu. Rev. Public Health **33**, 107-122. (10.1146/annurev-publhealth-031811-124655) 22429160 PMC3638991

[9] Reiner Jr RC et al. 2013 A systematic review of mathematical models of mosquito-borne pathogen transmission: 1970–2010. J. R Soc. Interface. **10**, 20120921. (10.1098/rsif.2012.0921) 23407571 PMC3627099

[10] Tobler WR. 1970 A computer movie simulating urban growth in the detroit region. Econ. Geogr. **46**(sup1), 234-240. (10.2307/143141)

[11] Lessler J, Azman AS, Grabowski MK, Salje H, Rodriguez-Barraquer I. 2016 Trends in the mechanistic and dynamic modeling of infectious diseases. Curr. Epidemiol. Rep. **3**, 212-222. (10.1007/s40471-016-0078-4) 32226711 PMC7100697

[12] Lessler J, Cummings DAT. 2016 Mechanistic models of infectious disease and their impact on public health. Am. J. Epidemiol. **183**, 415-422. (10.1093/aje/kww021) 26893297 PMC5006438

[13] Bzdok D, Krzywinski M, Altman N. 2017 Machine learning: a primer. Nat. Methods **14**, 1119-1120. (10.1038/nmeth.4526) 29664466 PMC5905345

[14] Moher D, Liberati A, Tetzlaff J, Altman DG, Prisma Group. 2009 Preferred reporting items for systematic reviews and meta-analyses: the PRISMA statement. PLoS Med. **6**, e1000097. (10.1371/journal.pmed.1000097) 19621072 PMC2707599

[15] European Centre for Disease Prevention and Control. 2020 Mosquito-borne diseases. European Centre for Disease Prevention and Control. https://www.ecdc.europa.eu/en/mosquito-borne-diseases (accessed 21 July 2020).

[16] World Health Organization. 2020 Mosquito-borne diseases. http://www.who.int/neglected_diseases/vector_ecology/mosquito-borne-diseases/en/ (accessed 21 July 2020).

[17] R Core Team. 2019 R: a language and environment for statistical computing. Vienna, Austria: R Foundation for Statistical Computing. See https://www.R-project.org.

[18] Lee SA. 2021 A data and R code to accompany ‘Spatial connectivity in mosquito-borne disease models: a systematic review of methods and assumptions' (version v1.0.0). Zenodo. (10.5281/zenodo.4706866) PMC8150046 34034534

[19] Bomfim R, Pei S, Shaman J, Yamana T, Makse HA, Andrade JS, Lima Neto AS, Furtado V. 2020 Predicting dengue outbreaks at neighbourhood level using human mobility in urban areas. J. R. Soc. Interface **17**, 20200691. (10.1098/rsif.2020.0691) 33109025 PMC7653379

[20] Chen Y, Yang Z, Jing Q, Huang J, Guo C, Yang K, Chen A, Lu J. 2020 Effects of natural and socioeconomic factors on dengue transmission in two cities of China from 2006 to 2017. Sci. Total Environ. **724**, 138200. (10.1016/j.scitotenv.2020.138200) 32408449

[21] Astutik S, Rahayudi B, Iskandar A, Fitriani R. 2013 Bayesian spatial-temporal autologistic regression model on dengue hemorrhagic fever in East Java, Indonesia. Appl. Math Sci. **7**, 435-443. (10.12785/amis/072L08)

[22] Wu PC, Lay JG, Guo HR, Lin CY, Lung SC, Su HJ. 2009 Higher temperature and urbanization affect the spatial patterns of dengue fever transmission in subtropical Taiwan. Sci. Total Environ. **407**, 2224-2233. (10.1016/j.scitotenv.2008.11.034) 19157509

[23] DeGroote JP, Sugumaran R. 2012 National and regional associations between human West Nile virus incidence and demographic, landscape, and land use conditions in the coterminous United States. Vector-Borne Zoonotic Dis. **12**, 657-665. (10.1089/vbz.2011.0786) 22607071

[24] Jain R, Sontisirikit S, Iamsirithaworn S, Prendinger H. 2019 Prediction of dengue outbreaks based on disease surveillance, meteorological and socio-economic data. BMC Infect. Dis. **19**, 272. (10.1186/s12879-019-3874-x) 30898092 PMC6427843

[25] Impoinvil DE, Solomon T, Schluter WW, Rayamajhi A, Bichha RP, Shakya G, Caminade C, Baylis M. 2011 The spatial heterogeneity between Japanese encephalitis incidence distribution and environmental variables in Nepal. PLoS ONE **6**, e22192. (10.1371/journal.pone.0022192) 21811573 PMC3141013

[26] Chuang TW, Ng KC, Nguyen TL, Chaves LF. 2018 Epidemiological characteristics and space-time analysis of the 2015 dengue outbreak in the metropolitan region of Tainan city, Taiwan. Int. J. Environ. Res. Public Health. **15**, 396. (10.3390/ijerph15030396) PMC5876941 29495351

[27] Wen T-H, Tsai C-T. 2016 Evaluating the role of disease importation in the spatiotemporal transmission of indigenous dengue outbreak. Appl. Geogr. **76**, 137-146. (10.1016/j.apgeog.2016.09.020)

[28] Xu Z, Bambrick H, Pongsumpun P, Tang IM, Yakob L, Devine G, Frentiu FD, Williams G, Hu W. 2020 Does Bangkok have a central role in the dengue dynamics of Thailand? Parasit. Vectors **13**, 1-9. (10.1186/s13071-019-3862-4) 31931886 PMC6958813

[29] Gunderson AK et al. 2020 Malaria transmission and spillover across the Peru–Ecuador border: a spatiotemporal analysis. Int. J. Environ. Res. Public Health **17**, 7434. (10.3390/ijerph17207434) PMC7600436 33066022

[30] Ashmore P, Lindahl JF, Colón-González FJ, Sinh Nam V, Quang Tan D, Medley GF. 2020 Spatiotemporal and socioeconomic risk factors for dengue at the province level in Vietnam, 2013–2015: clustering analysis and regression model. Trop. Med. Infect. Dis. **5**, 81. (10.3390/tropicalmed5020081) PMC7345007 32438628

[31] Tao H, Wang K, Zhuo L, Li X, Li Q, Liu Y, Xu Y. 2019 A comprehensive framework for studying diffusion patterns of imported dengue with individual-based movement data. Int. J. Geogr. Inf. Sci. **34**, 604-624. (10.1080/13658816.2019.1684497)

[32] Kraemer MUG et al. 2018 Inferences about spatiotemporal variation in dengue virus transmission are sensitive to assumptions about human mobility: a case study using geolocated tweets from Lahore. Pakistan. Epj Data Sci. **7**, 1-7. (10.1140/epjds/s13688-018-0144-x) PMC6404370 30854281

[33] Salami D, Capinha C, Martins MdR, Sousa CA. 2020 Dengue importation into Europe: a network connectivity-based approach. PLoS ONE **15**, e0230274. (10.1371/journal.pone.0230274) 32163497 PMC7067432

[34] Ramadona AL, Tozan Y, Lazuardi L, Rocklöv J. 2019 A combination of incidence data and mobility proxies from social media predicts the intra-urban spread of dengue in Yogyakarta, Indonesia. PLoS Negl. Trop. Dis. **13**, e0007298. (10.1371/journal.pntd.0007298) 30986218 PMC6483276

[35] Cauchemez S, Ledrans M, Poletto C, Quénel P, De Valk H, Colizza V, Boëlle PY. 2014 Local and regional spread of chikungunya fever in the Americas. Eurosurveillance **19**, 20854. (10.2807/1560-7917.ES2014.19.28.20854) 25060573 PMC4340072

[36] Kraemer MU et al. 2017 Spread of yellow fever virus outbreak in Angola and the Democratic Republic of the Congo 2015–16: a modelling study. Lancet Infect. Dis. **17**, 330-338. (10.1016/S1473-3099(16)30513-8) 28017559 PMC5332542

[37] Da Silva-Nunes M, Codeço CT, Malafronte RS, Da Silva NS, Juncansen C, Muniz PT, Da Silva NS, Da Silva-Nunes M. 2008 Malaria on the Amazonian frontier: transmission dynamics, risk factors, spatial distribution, and prospects for control. Am. J. Trop. Med. Hyg. **79**, 624-635. (10.4269/ajtmh.2008.79.624) 18840755

[38] Parra MCP, Fávaro EA, Dibo MR, Mondini A, Eiras ÁE, Kroon EG, Teixeira MM, Nogueira ML, Chiaravalloti-Neto F. 2018 Using adult *Aedes aegypti* females to predict areas at risk for dengue transmission: a spatial case–control study. Acta Trop. **182**, 43-53. (10.1016/j.actatropica.2018.02.018) 29462598

[39] Wang X, Su L, Zhu H, Hu W, An J, Wang C, Qiannan E, Qi X, Zhuang G. 2020 Long-term epidemiological dynamics of Japanese encephalitis infection in Gansu Province, China: a spatial and temporal analysis. Am. J. Trop. Med. Hyg. **103**, 2065-2076. (10.4269/ajtmh.20-0179) 32996458 PMC7646783

[40] Kazembe LN. 2007 Spatial modelling and risk factors of malaria incidence in northern Malawi. Acta Trop. **102**, 126-137. (10.1016/j.actatropica.2007.04.012) 17543264

[41] Gomes MFC, Codeço CT, Bastos LS, Lana RM. 2020 Measuring the contribution of human mobility to malaria persistence. Malar. J. **19**, 404. (10.1186/s12936-020-03474-4) 33176792 PMC7659106

[42] Griffith DA. 2005 A comparison of six analytical disease mapping techniques as applied to West Nile virus in the coterminous United States. Int. J. Health Geogr. **4**, 18. (10.1186/1476-072X-4-18) 16076391 PMC1215506

[43] Tevie J, Bohara A, Valdez RB. 2014 Examination of the geographical variation in human West Nile virus: a spatial filtering approach. Epidemiol. Infect. **142**, 2522-2529. (10.1017/S0950268814000090) 24512765 PMC9151312

[44] Kim S, Kim Y. 2019 Spatially filtered multilevel analysis on spatial determinants for malaria occurrence in Korea. Int. J. Environ. Res. Public Health **16**, 11. (10.3390/ijerph16071250) PMC6480462 30965608

[45] Griffith DA. 2000 A linear regression solution to the spatial autocorrelation problem. J. Geogr. Syst. **2**, 141-156. (10.1007/PL00011451)

[46] Prem K, Lau MSY, Tam CC, Ho MZJ, Ng LC, Cook AR. 2019 Inferring who-infected-whom-where in the 2016 Zika outbreak in Singapore—a spatio-temporal model. J. R Soc. Interface **16**, 20180604. (10.1098/rsif.2018.0604) 31213175 PMC6597776

[47] Delmelle E, Hagenlocher M, Kienberger S, Casas I. 2016 A spatial model of socioeconomic and environmental determinants of dengue fever in Cali, Colombia. Acta Trop. **164**, 169-176. (10.1016/j.actatropica.2016.08.028) 27619189

[48] Robertson C, Pant DK, Joshi DD, Sharma M, Dahal M, Stephen C. 2013 Comparative spatial dynamics of Japanese encephalitis and acute encephalitis syndrome in Nepal. PLoS ONE **8**, e66168. (10.1371/journal.pone.0066168) 23894277 PMC3718805

[49] Ehlkes L et al. 2014 Geographically weighted regression of land cover determinants of *Plasmodium falciparum* transmission in the Ashanti Region of Ghana. Int. J. Health Geogr. **13**, 1-11. (10.1186/1476-072X-13-35) 25270342 PMC4192530

[50] Atique S, Chan TC, Chen CC, Hsu CY, Iqtidar S, Louis VR, Shabbir SA, Chuang T-W. 2018 Investigating spatio-temporal distribution and diffusion patterns of the dengue outbreak in Swat, Pakistan. J. Infect. Public Health **11**, 550-557. (10.1016/j.jiph.2017.12.003) 29287804

[51] Manyangadze T, Chimbari MJ, Macherera M, Mukaratirwa S. 2017 Micro-spatial distribution of malaria cases and control strategies at ward level in Gwanda district, Matabeleland South, Zimbabwe. Malar. J. **16**, 1-11. (10.1186/s12936-017-2116-1) 29162102 PMC5697109

[52] Khormi HM, Kumar L. 2011 Modeling dengue fever risk based on socioeconomic parameters, nationality and age groups: GIS and remote sensing based case study. Sci. Total Environ. **409**, 4713-4719. (10.1016/j.scitotenv.2011.08.028) 21906782

[53] Acharya BK, Cao CX, Lakes T, Chen W, Naeem S, Pandit S. 2018 Modeling the spatially varying risk factors of dengue fever in Jhapa district, Nepal, using the semi-parametric geographically weighted regression model. Int. J. Biometeorol. **62**, 1973-1986. (10.1007/s00484-018-1601-8) 30182200

[54] Hasyim H, Nursafingi A, Haque U, Montag D, Groneberg DA, Dhimal M, Kuch U, Müller R. 2018 Spatial modelling of malaria cases associated with environmental factors in South Sumatra, Indonesia. Malar. J. **17**, 1-15. (10.1186/s12936-017-2149-5) 29463239 PMC5819714

[55] Homan T et al. 2016 Spatially variable risk factors for malaria in a geographically heterogeneous landscape, western Kenya: an explorative study. Malar. J. **15**, 1. (10.1186/s12936-015-1044-1) 26729363 PMC4700570

[56] Ren H, Wu W, Li T, Yang Z. 2019 Urban villages as transfer stations for dengue fever epidemic: a case study in the Guangzhou, China. PLoS Negl. Trop. Dis. **13**, e0007350. (10.1371/journal.pntd.0007350) 31022198 PMC6504109

[57] Lin C-H, Wen T-H. 2011 Using geographically weighted regression (GWR) to explore spatial varying relationships of immature mosquitoes and human densities with the incidence of dengue. Int. J. Environ. Res. Public Health **8**, 2798-2815. (10.3390/ijerph8072798) 21845159 PMC3155330

[58] Grillet ME, Barrera R, Martínez JE, Berti J, Fortin MJ. 2010 Disentangling the effect of local and global spatial variation on a mosquito-borne infection in a neotropical heterogeneous environment. Am. J. Trop. Med. Hyg. **82**, 194-201. (10.4269/ajtmh.2010.09-0040) 20133991 PMC2813156

[59] Yang D, Xu C, Wang J, Zhao Y. 2017 Spatiotemporal epidemic characteristics and risk factor analysis of malaria in Yunnan Province, China. BMC Public Health **17**, 1-10. (10.1186/s12889-016-3954-4) 28077125 PMC5225622

[60] Gopal S, Ma Y, Xin C, Pitts J, Were L. 2019 Characterizing the spatial determinants and prevention of malaria in Kenya. Int. J. Environ. Res. Public Health. **16**, 5078. (10.3390/ijerph16245078) PMC6950158 31842408

[61] de Oliveira Padilha MA, de Oliveira Melo J, Romano G, de Lima MVM, Alonso WJ, Sallum MAM, Laporta GZ. 2019 Comparison of malaria incidence rates and socioeconomic–environmental factors between the states of Acre and Rondonia: a spatio-temporal modelling study. Malar. J. **18**, 04. (10.1186/s12936-019-2938-0) PMC6727495 31484519

[62] Ren H, Zheng L, Li Q, Yuan W, Lu L. 2017 Exploring determinants of spatial variations in the dengue fever epidemic using geographically weighted regression model: a case study in the joint Guangzhou–Foshan area, China, 2014. Int. J. Environ. Res. Public Health **14**, 1518. (10.3390/ijerph14121518) PMC5750936 29211001

[63] Halimi M, Farajzadeh M, Delavari M, Takhtardeshir A, Moradi A. 2014 Modelling spatial relationship between climatic conditions and annual parasite incidence of malaria in southern part of Sistan&Balouchistan Province of Iran using spatial statistic models. Asian Pac. J. Trop. Dis. **4**(Suppl. 1), S167-S172. (10.1016/S2222-1808(14)60434-5)

[64] Ge Y, Song Y, Wang J, Liu W, Ren Z, Peng J, Lu B. 2017 Geographically weighted regression-based determinants of malaria incidences in northern China. Trans. GIS **21**, 934-953. (10.1111/tgis.12259)

[65] Anjos Rd, Nóbrega RS, Ferreira HdS, Lacerda Ad, Sousa-Neves Nd. 2020 Exploring local and global regression models to estimate the spatial variability of Zika and chikungunya cases in Recife, Brazil. Rev. Soc. Bras. Med. Trop. **53**, e20200027. (10.1590/0037-8682-0027-2020) 32997047 PMC7523520

[66] Brunsdon C, Fotheringham S, Charlton M. 1998 Geographically weighted regression. J. R. Stat. Soc. Ser. Stat. **47**, 431-443. (10.1111/1467-9884.00145) 16118814

[67] Samat N, Percy D. 2012 Vector-borne infectious disease mapping with stochastic difference equations: an analysis of dengue disease in Malaysia. J. Appl. Stat. **39**, 2029-2046. (10.1080/02664763.2012.700450)

[68] Flórez-Lozano K et al. 2020 Spatial distribution of the relative risk of Zika virus disease in Colombia during the 2015–2016 epidemic from a Bayesian approach. Int. J. Gynecol. Obstet. **148**(S2), 55-60. (10.1002/ijgo.13048) PMC7065154 31975401

[69] Ferreira GS, Schmidt AM. 2006 Spatial modelling of the relative risk of dengue fever in Rio de Janeiro for the epidemic period between 2001 and 2002. Braz. J. Probab. Stat. **20**, 29-47.

[70] Noor AM, Kinyoki DK, Mundia CW, Kabaria CW, Mutua JW, Alegana VA, Fall IS, Snow RW. 2014 The changing risk of *Plasmodium falciparum* malaria infection in Africa: 2000–10: A spatial and temporal analysis of transmission intensity. Lancet **383**, 1739-1747. (10.1016/S0140-6736(13)62566-0) 24559537 PMC4030588

[71] Ssempiira J et al. 2018 The effect of case management and vector-control interventions on space-time patterns of malaria incidence in Uganda. Malar. J. 2018/04/14 ed. **17**, 162. (10.1186/s12936-018-2312-7) 29650005 PMC5898071

[72] Wangdi K, Canavati SE, Ngo TD, Tran LK, Nguyen TM, Tran DT, Martin NJ, Clements ACA. 2018 Analysis of clinical malaria disease patterns and trends in Vietnam 2009–2015. Malar. J. **17**, 15. (10.1186/s12936-018-2478-z) 30223843 PMC6142383

[73] Villalta D, Guenni L, Rubio-Palis Y, Ramírez Arbeláez R. 2013 Bayesian space-time modeling of malaria incidence in Sucre state, Venezuela. AStA Adv. Stat. Anal. **97**, 151-171. (10.1007/s10182-012-0190-9)

[74] Jaya I, Abdullah AS, Hermawan E, Ruchjana BN. 2016 Bayesian spatial modeling and mapping of dengue fever: a case study of dengue fever in the city of Bandung. Indonesia. Int. J. Appl. Math Stat. **54**, 94-103.

[75] Thway AM, Rotejanaprasert C, Sattabongkot J, Lawawirojwong S, Thi A, Hlaing TM, Soe TM, Kaewkungwal J. 2018 Bayesian spatiotemporal analysis of malaria infection along an international border: Hlaingbwe Township in Myanmar and Tha-Song-Yang District in Thailand. Malar. J. **17**, 428. (10.1186/s12936-018-2574-0) 30445962 PMC6240260

[76] Jaya I, Folmer H. 2020 Bayesian spatiotemporal mapping of relative dengue disease risk in Bandung, Indonesia. J. Geogr. Syst. **22**, 105-142. (10.1007/s10109-019-00311-4)

[77] Rouamba T, Samadoulougou S, Tinto H, Alegana VA, Kirakoya-Samadoulougou F. 2020 Bayesian spatiotemporal modeling of routinely collected data to assess the effect of health programs in malaria incidence during pregnancy in Burkina Faso. Sci. Rep. **10**, 14. (10.1038/s41598-019-56010-z) 32060297 PMC7021681

[78] Rotejanaprasert C, Ekapirat N, Areechokchai D, Maude RJ. 2020 Bayesian spatiotemporal modeling with sliding windows to correct reporting delays for real-time dengue surveillance in Thailand. Int. J. Health Geogr. **19**, 1-13. (10.1186/s12942-020-00199-0) 32126997 PMC7055098

[79] Snow RW, Kibuchi E, Karuri SW, Sang G, Gitonga CW, Mwandawiro C, Bejon P, Noor AM. 2015 Changing malaria prevalence on the Kenyan coast since 1974: climate, drugs and vector control. PLoS ONE **10**, e0128792. (10.1371/journal.pone.0128792) 26107772 PMC4479373

[80] Reid HL, Haque U, Roy S, Islam N, Clements ACA. 2012 Characterizing the spatial and temporal variation of malaria incidence in Bangladesh, 2007. Malar. J. **11**, 170. (10.1186/1475-2875-11-170) 22607348 PMC3465176

[81] Aswi A, Cramb S, Duncan E, Hu W, White G, Mengersen K. 2020 Climate variability and dengue fever in Makassar, Indonesia: Bayesian spatio-temporal modelling. Spat. Spatio-Temporal Epidemiol. **33**, 100335. (10.1016/j.sste.2020.100335) 32370940

[82] Lowe R et al. 2014 Dengue outlook for the World Cup in Brazil: an early warning model framework driven by real-time seasonal climate forecasts. Lancet Infect. Dis. **14**, 619-626. (10.1016/S1473-3099(14)70781-9) 24841859

[83] Wang G, Minnis RB, Belant JL, Wax CL. 2010 Dry weather induces outbreaks of human West Nile virus infections. BMC Infect. Dis. **10**, 38. (10.1186/1471-2334-10-38) 20181272 PMC2841181

[84] Wimberly MC, Hildreth MB, Boyte SP, Lindquist E, Kightlinger L. 2008 Ecological niche of the 2003 West Nile virus epidemic in the northern Great Plains of the United States. PLoS ONE **3**, e3744. (10.1371/journal.pone.0003744) 19057643 PMC2586649

[85] Ouédraogo M, Rouamba T, Samadoulougou S, Kirakoya-Samadoulougou F. 2020 Effect of free healthcare policy for children under five years old on the incidence of reported malaria cases in burkina faso by bayesian modelling: ‘not only the ears but also the head of the hippopotamus’. Int. J. Environ. Res. Public Health **17**, 417. (10.3390/ijerph17020417) PMC7014427 31936308

[86] Umer MF, Zofeen S, Majeed A, Hu W, Qi X, Zhuang G. 2019 Effects of socio-environmental factors on malaria infection in Pakistan: a Bayesian spatial analysis. Int. J. Environ. Res. Public Health **16**, 1365. (10.3390/ijerph16081365) PMC6517989 30995744

[87] Abd Naeeim NS, Rahman NA. 2017 Estimating relative risk for dengue disease in Peninsular Malaysia using INLA. Malays. J. Fundam. Appl. Sci. **13**, 721-727. (10.11113/mjfas.v0n0.575)

[88] Alegana VA, Atkinson PM, Wright JA, Kamwi R, Uusiku P, Katokele S, Snow RW, Noor AM. 2013 Estimation of malaria incidence in northern Namibia in 2009 using Bayesian conditional-autoregressive spatial-temporal models. Spat. Spatio-Temporal Epidemiol. **7**, 25-36. (10.1016/j.sste.2013.09.001) PMC3839406 24238079

[89] Mukhsar, Abapihi B, Sani A, Cahyono E, Adam P, Abdullah FA. 2016 Extended convolution model to bayesian spatio-temporal for diagnosing the DHF endemic locations. J. Interdiscip. Math. **19**, 233-244. (10.1080/09720502.2015.1047591)

[90] Husnina Z, Clements ACA, Wangdi K. 2019 Forest cover and climate as potential drivers for dengue fever in Sumatra and Kalimantan 2006–2016: a spatiotemporal analysis. Trop. Med. Int. Health **24**, 888-898. (10.1111/tmi.13248) 31081162

[91] Lekdee K, Ingsrisawang L. 2013 Generalized linear mixed models with spatial random effects for spatio-temporal data: an application to dengue fever mapping. J. Math. Stat. **9**, 137-143. (10.3844/jmssp.2013.137.143)

[92] Nkurunziza H, Gebhardt A, Pilz J. 2011 Geo-additive modelling of malaria in Burundi. Malar. J. **10**, 234. (10.1186/1475-2875-10-234) 21835010 PMC3180443

[93] Zayeri F, Salehi M, Pirhosseini H. 2011 Geographical mapping and Bayesian spatial modeling of malaria incidence in Sistan and Baluchistan province, Iran. Asian Pac. J. Trop. Med. **4**, 985-992. (10.1016/S1995-7645(11)60231-9) 22118036

[94] Chien LC, Yu HL. 2014 Impact of meteorological factors on the spatiotemporal patterns of dengue fever incidence. Environ. Int. **73**, 46-56. (10.1016/j.envint.2014.06.018) 25084561

[95] Ssempiira J, Kissa J, Nambuusi B, Mukooyo E, Opigo J, Makumbi F, Kasasa S, Vounatsou P. 2018 Interactions between climatic changes and intervention effects on malaria spatio-temporal dynamics in Uganda. Parasit. Epidemiol. Control **3**, e00070. (10.1016/j.parepi.2018.e00070) PMC6020080 29988311

[96] Zhao X, Cao M, Feng HH, Fan H, Chen F, Feng Z, Li X, Zhou X-H. 2014 Japanese encephalitis risk and contextual risk factors in Southwest China: a Bayesian hierarchical spatial and spatiotemporal analysis. Int. J. Environ. Res. Public Health **11**, 4201-4217. (10.3390/ijerph110404201) 24739769 PMC4024990

[97] Martínez-Bello DA, López-Quílez A, Prieto AT. 2019 Joint estimation of relative risk for dengue and Zika infections, Colombia, 2015–2016. Emerg. Infect. Dis. **25**, 1118-1126. (10.3201/eid2506.180392) 31107226 PMC6537708

[98] Zacarias OP, Andersson M. 2010 Mapping malaria incidence distribution that accounts for environmental factors in Maputo Province - Mozambique. Malar. J. **9**, 79. (10.1186/1475-2875-9-79) 20302674 PMC2853555

[99] Huang F, Zhou S, Zhang S, Zhang H, Li W. 2011 Meteorological factors-based spatio-temporal mapping and predicting malaria in central China. Am. J. Trop. Med. Hyg. **85**, 560-567. (10.4269/ajtmh.2011.11-0156) 21896823 PMC3163885

[100] Alegana VA, Wright JA, Nahzat SM, Butt W, Sediqi AW, Habib N, Snow RW, Atkinson PM, Noor AM. 2014 Modelling the incidence of *Plasmodium vivax* and *Plasmodium falciparum* malaria in Afghanistan 2006–2009. PLoS ONE **9**, e102304. (10.1371/journal.pone.0102304) 25033452 PMC4102516

[101] Restrepo AC, Baker P, Clements ACA. 2014 National spatial and temporal patterns of notified dengue cases, Colombia 2007–2010. Trop. Med. Int. Health **19**, 863-871. (10.1111/tmi.12325) 24862214

[102] Santos-Vega M, Bouma MJ, Kohli V, Pascual M. 2016 Population density, climate variables and poverty synergistically structure spatial risk in Urban Malaria in India. PLoS Negl. Trop. Dis. **10**, e0005155. (10.1371/journal.pntd.0005155) 27906962 PMC5131912

[103] Lowe R, Cazelles B, Paul R, Rodó X. 2016 Quantifying the added value of climate information in a spatio-temporal dengue model. Stoch. Environ. Res. Risk Assess. **30**, 2067-2078. (10.1007/s00477-015-1053-1)

[104] Lowe R, Chirombo J, Tompkins AM. 2013 Relative importance of climatic, geographic and socio-economic determinants of malaria in Malawi. Malar. J. **12**, 416. (10.1186/1475-2875-12-416) 24228784 PMC4225758

[105] Sani A, Abapihi B, Mukhsar M, Kadir K. 2015 Relative risk analysis of dengue cases using convolution extended into spatio-temporal model. J. Appl. Stat. **42**, 2509-2519. (10.1080/02664763.2015.1043863)

[106] Samat NA, Pei Zhen W. 2017 Relative risk estimation for dengue disease mapping in Malaysia based on Besag, York and Mollié model. Pertanika J. Sci. Technol. **25**, 759-766.

[107] Martínez-Bello DA, López-Quílez A, Torres Prieto A. 2017 Relative risk estimation of dengue disease at small spatial scale. Int. J. Health Geogr. **16**, 31. (10.1186/s12942-017-0104-x) 28810908 PMC5558735

[108] Kristiani F, Yong B, Irawan R. 2016 Relative risk estimation of dengue disease in Bandung, Indonesia, using Poisson-gamma and bym models considering the severity level. J. Teknol. **78**, 57-64.

[109] Rouamba T, Samadoulougou S, Tinto H, Alegana VA, Kirakoya-Samadoulougou F. 2020 Severe-malaria infection and its outcomes among pregnant women in Burkina Faso health-districts: hierarchical Bayesian space-time models applied to routinely-collected data from 2013 to 2018. Spat. Spatio-Temporal Epidemiol. **33**, 100333. (10.1016/j.sste.2020.100333) PMC7613547 32370941

[110] Manh BH et al. 2011 Social and environmental determinants of malaria in space and time in Viet Nam. Int. J. Parasitol. **41**, 109-116. (10.1016/j.ijpara.2010.08.005) 20833173 PMC3086784

[111] Clements AC, Barnett AG, Cheng ZW, Snow RW, Zhou HN. 2009 Space-time variation of malaria incidence in Yunnan province, China. Malar. J. 2009/08/04 ed. **8**, 180. (10.1186/1475-2875-8-180) 19646240 PMC2724544

[112] de Almeida AS, de Andrade Medronho R, Ortiz Valencia LI. 2009 Spatial analysis of dengue and the socioeconomic context of the city of Rio de Janeiro (Southeastern Brazil). Rev. Saude Publica **43**, 666-673. (10.1590/S0034-89102009000400013) 19649472

[113] Honorato T, Lapa PPA, Sales CMM, Reis-Santos B, Tristão-Sá R, Bertolde AI, Maciel ELN. 2014 Spatial analysis of distribution of dengue cases in Espírito Santo, Brazil, in 2010: use of Bayesian model. Rev. Bras. Epidemiol. **17**, 150-159. (10.1590/1809-4503201400060013) 25409645

[114] Noh M, Lee Y, Oh S, Chu C, Gwack J, Youn SK, Cho SH, Lee WJ, Huh S. 2012 Spatial and temporal distribution of *Plasmodium vivax* malaria in Korea estimated with a hierarchical generalized linear model. Osong Public Health Res. Perspect. **3**, 192-198. (10.1016/j.phrp.2012.11.003) 24159514 PMC3747662

[115] Phanitchat T et al. 2019 Spatial and temporal patterns of dengue incidence in northeastern Thailand 2006–2016. BMC Infect. Dis. **19**, 743. (10.1186/s12879-019-4379-3) 31443630 PMC6708185

[116] Wangdi K, Clements ACA, Du T, Nery SV. 2018 Spatial and temporal patterns of dengue infections in Timor-Leste, 2005–2013. Parasit. Vectors **11**, 9. (10.1186/s13071-017-2588-4) 29301546 PMC5755460

[117] Ouédraogo M, Samadoulougou S, Rouamba T, Hien H, Sawadogo JEM, Tinto H, Alegana VA, Speybroeck N, Kirakoya-Samadoulougou F. 2018 Spatial distribution and determinants of asymptomatic malaria risk among children under 5 years in 24 districts in Burkina Faso. Malar. J. **17**, 460. (10.1186/s12936-018-2606-9) 30526598 PMC6286519

[118] Kikuti M et al. 2015 Spatial distribution of dengue in a Brazilian Urban slum setting: role of socioeconomic gradient in disease risk. PLoS Negl. Trop. Dis. **9**, e0003937. (10.1371/journal.pntd.0003937) 26196686 PMC4510880

[119] Costa JV, Donalisio MR, Silveira LdA. 2013 Spatial distribution of dengue incidence and socio-environmental conditions in Campinas, Sao Paulo State, Brazil, 2007. Cad. Saude Publica **29**, 1522-1532. (10.1590/S0102-311X2013001200005) 24005918

[120] Teixeira TdA, Cruz OG. 2011 Spatial modeling of dengue and socio-environmental indicators in the city of Rio de Janeiro, Brazil. Cad. Saude Publica **27**, 591-602. (10.1590/S0102-311X2011000300019) 21519709

[121] Hu WB, Clements A, Williams G, Tong SL, Mengersen K. 2012 Spatial patterns and socioecological drivers of dengue fever transmission in Queensland, Australia. Environ. Health Perspect. **120**, 260-266. (10.1289/ehp.1003270) 22015625 PMC3279430

[122] Abellana R, Ascaso C, Aponte J, Saute F, Nhalungo D, Nhacolo A, Alonso P. 2008 Spatio-seasonal modeling of the incidence rate of malaria in Mozambique. Malar. J. **7**, 228. (10.1186/1475-2875-7-228) 18976458 PMC2584655

[123] Mabaso MLH, Vounatsou P, Midzi S, Da Silva J, Smith T. 2006 Spatio-temporal analysis of the role of climate in inter-annual variation of malaria incidence in Zimbabwe. Int. J. Health Geogr. **5**, 20. (10.1186/1476-072X-5-20) 16700905 PMC1513195

[124] Martínez-Bello DA, López-Quílez A, Torres Prieto A. 2018 Spatio-temporal modeling of Zika and dengue infections within Colombia. Int. J. Environ. Res. Public Health **15**, 1376. (10.3390/ijerph15071376) PMC6068969 29966348

[125] Lowe R, Bailey TC, Stephenson DB, Graham RJ, Coelho CAS, Sá Carvalho M, Barcellos C. 2011 Spatio-temporal modelling of climate-sensitive disease risk: towards an early warning system for dengue in Brazil. Comput. Geosci. **37**, 371-381. (10.1016/j.cageo.2010.01.008)

[126] Nobre AA, Schmidt AM, Lopes HF. 2005 Spatio-temporal models for mapping the incidence of malaria in Pará. Environmetrics **16**, 291-304. (10.1002/env.704)

[127] Bett B et al. 2019 Spatiotemporal analysis of historical records (2001–2012) on dengue fever in Vietnam and development of a statistical model for forecasting risk. PLoS ONE **14**, e0224353. (10.1371/journal.pone.0224353) 31774823 PMC6881000

[128] McHale TC, Romero-Vivas CM, Fronterre C, Arango-Padilla P, Waterlow NR, Nix CD, Falconar AK, Cano J. 2019 Spatiotemporal heterogeneity in the distribution of chikungunya and Zika virus case incidences during their 2014 to 2016 epidemics in Barranquilla, Colombia. Int. J. Environ. Res. Public Health **16**, 1759. (10.3390/ijerph16101759) PMC6572372 31109024

[129] Martínez-Bello D, López-Quílez A, Prieto AT. 2018 Spatiotemporal modeling of relative risk of dengue disease in Colombia. Stoch. Environ. Res. Risk Assess. **32**, 1587-1601. (10.1007/s00477-017-1461-5)

[130] Chien L, Lin R, Liao Y, Sy F, Perez A. 2018 Surveillance on the endemic of Zika virus infection by meteorological factors in Colombia: a population-based spatial and temporal study. BMC Infect. Dis. **18**, 180. (10.1186/s12879-018-3085-x) 29665783 PMC5905128

[131] Lowe R, Bailey TC, Stephenson DB, Jupp TE, Graham RJ, Barcellos C, Carvalho M. 2013 The development of an early warning system for climate-sensitive disease risk with a focus on dengue epidemics in Southeast Brazil. Stat. Med. **32**, 864-883. (10.1002/sim.5549) 22927252

[132] Wijayanti SPM, Porphyre T, Chase-Topping M, Rainey SM, McFarlane M, Schnettler E, Biek R, Kohl A. 2016 The importance of socio-economic versus environmental risk factors for reported dengue cases in Java, Indonesia. PLoS Negl. Trop. Dis. **10**, 15. (10.1371/journal.pntd.0004964) PMC5014450 27603137

[133] Mabaso MLH, Craig M, Vounatsou P, Smith T. 2005 Towards empirical description of malaria seasonality in southern Africa: the example of Zimbabwe. Trop. Med. Int. Health **10**, 909-918. (10.1111/j.1365-3156.2005.01462.x) 16135199

[134] Adin A, Martínez-Bello DA, López-Quílez A, Ugarte MD. 2018 Two-level resolution of relative risk of dengue disease in a hyperendemic city of Colombia. PLoS ONE **13**, e0203382. (10.1371/journal.pone.0203382) 30204762 PMC6133285

[135] Achcar JA, Martinez EZ, Souza AD, Tachibana VM, Flores EF. 2011 Use of Poisson spatiotemporal regression models for the Brazilian Amazon Forest: malaria count data. Rev. Soc. Bras. Med. Trop. 2012/01/11 ed. **44**, 749-754. (10.1590/S0037-86822011000600019) 22231249

[136] Hanandita W, Tampubolon G. 2016 Geography and social distribution of malaria in Indonesian Papua: a cross-sectional study. Int. J. Health Geogr. **15**, 13. (10.1186/s12942-016-0043-y) 27072128 PMC4830039

[137] Zhang SB, Hu WB, Qi X, Zhuang GH. 2018 How socio-environmental factors are associated with Japanese encephalitis in Shaanxi, China—a Bayesian spatial analysis. Int. J. Environ. Res. Public Health **15**, 13. (10.3390/ijerph15040608) PMC5923650 29584661

[138] Battle KE et al. 2019 Mapping the global endemicity and clinical burden of *Plasmodium vivax*, 2000–17: a spatial and temporal modelling study. Lancet **394**, 332-343. (10.1016/S0140-6736(19)31096-7) 31229233 PMC6675736

[139] Yu HL, Lee CH, Chien LC. 2016 A spatiotemporal dengue fever early warning model accounting for nonlinear associations with hydrological factors: a Bayesian maximum entropy approach. Stoch. Environ. Res. Risk Assess. **30**, 2127-2141. (10.1007/s00477-016-1328-1)

[140] Maheu-Giroux M, Castro MC. 2013 Impact of community-based larviciding on the prevalence of malaria infection in Dar es Salaam, Tanzania. PLoS ONE **8**, e71638. (10.1371/journal.pone.0071638) 23977099 PMC3743749

[141] Abd Naeeim NS, Rahman NA, Fahimi FAM. 2020 A spatial-temporal study of dengue in Peninsular Malaysia for the year 2017 in two different space–time model. J. Appl. Stat. **47**, 739-756. (10.1080/02664763.2019.1648391) PMC9041983 35707492

[142] Kleinschmidt I, Sharp B, Mueller I, Vounatsou P. 2002 Rise in malaria incidence rates in South Africa: a small-area spatial analysis of variation in time trends. Am. J. Epidemiol. **155**, 257-264. (10.1093/aje/155.3.257) 11821251

[143] Bisanzio D, Mutuku F, LaBeaud AD, Mungai PL, Muinde J, Busaidy H, Mukoko D, King CH, Kitron U. 2015 Use of prospective hospital surveillance data to define spatiotemporal heterogeneity of malaria risk in coastal Kenya. Malar. J. **14**, 482. (10.1186/s12936-015-1006-7) 26625721 PMC4665820

[144] Nakhapakorn K, Sancharoen W, Mutchimwong A, Jirakajohnkool S, Onchang R, Rotejanaprasert C, Tantrakarnapa K, Paul R. 2020 Assessment of urban land surface temperature and vertical city associated with dengue incidences. Remote Sens. **12**, 3802. (10.3390/rs12223802)

[145] Semakula M, Niragire F, Faes C. 2020 Bayesian spatio-temporal modeling of malaria risk in Rwanda. PLoS ONE **15**, e0238504. (10.1371/journal.pone.0238504) 32911503 PMC7482939

[146] Akter R, Hu W, Gatton M, Bambrick H, Cheng J, Tong S. 2020 Climate variability, socio-ecological factors and dengue transmission in tropical Queensland, Australia: a Bayesian spatial analysis. Environ. Res. **195**, 110285. (10.1016/j.envres.2020.110285) 33027631

[147] Kristiani F, Claudia Y, Yong B, Hilsdon A-M. 2020 A comparative analysis of frequentist and Bayesian approaches to estimate dengue disease transmission in Bandung-Indonesia. J. Stat. Manag. Syst. **23**, 1543-1559. (10.1080/09720510.2020.1756049)

[148] Ye J, Moreno-Madriñán MJ. 2020 Comparing different spatio-temporal modeling methods in dengue fever data analysis in Colombia during 2012–2015. Spat. Spatio-Temporal Epidemiol. **34**, 100360. (10.1016/j.sste.2020.100360) 32807397

[149] da Conceição Araújo D, Dos Santos AD, Lima SVMA, Vaez AC, Cunha JO, de Araújo KCGM. 2020 Determining the association between dengue and social inequality factors in north-eastern Brazil: a spatial modelling. Geospat. Health **15**, 71-80. (10.4081/gh.2020.854) 32575962

[150] Aswi A, Cramb S, Duncan E, Mengersen K. 2020 Evaluating the impact of a small number of areas on spatial estimation. Int. J. Health Geogr. **19**, 39. (10.1186/s12942-020-00233-1) 32977803 PMC7519538

[151] Jaya IGNM, Folmer H. 2020 Identifying spatiotemporal clusters by means of agglomerative hierarchical clustering and Bayesian regression analysis with spatiotemporally varying coefficients: methodology and application to dengue disease in Bandung, Indonesia. Geogr. Anal. (10.1111/gean.12264)

[152] Tsheten T, Clements ACA, Gray DJ, Wangchuk S, Wangdi K. 2020 Spatial and temporal patterns of dengue incidence in Bhutan: a Bayesian analysis. Emerg. Microbes Infect. **9**, 1360-1371. (10.1080/22221751.2020.1775497) 32538299 PMC7473275

[153] Wangdi K, Canavati SE, Ngo TD, Nguyen TM, Tran LK, Kelly GC, Martin NJ, Clements ACA. 2020 Spatial and temporal patterns of Malaria in Phu Yen Province, Vietnam, from 2005 to 2016. Am. J. Trop. Med. Hyg. **103**, 1540-1548. (10.4269/ajtmh.20-0392) 32748781 PMC7543816

[154] Wangdi K, Xu Z, Suwannatrai AT, Kurscheid J, Lal A, Namgay R, Glass K, Gray DJ, Clements ACA. 2020 A spatio-temporal analysis to identify the drivers of malaria transmission in Bhutan. Sci. Rep. **10**, 7060. (10.1038/s41598-020-63896-7) 32341415 PMC7184595

[155] Carabali M, Harper S, Lima Neto AS, Dos Santos de Sousa G, Caprara A, Restrepo BN, Kaufman JS. 2020 Spatiotemporal distribution and socioeconomic disparities of dengue, chikungunya and Zika in two Latin American cities from 2007 to 2017. Trop. Med. Int. Health **26**, 301-315. (10.1111/tmi.13530) 33219561

[156] Puggioni G, Couret J, Serman E, Akanda AS, Ginsberg HS. 2020 Spatiotemporal modeling of dengue fever risk in Puerto Rico. Spat. Spatio-Temporal Epidemiol. **35**, 100375. (10.1016/j.sste.2020.100375) 33138945

[157] Mallya S, Sander B, Roy-Gagnon MH, Taljaard M, Jolly A, Kulkarni MA. 2018 Factors associated with human West Nile virus infection in Ontario: a generalized linear mixed modelling approach. BMC Infect. Dis. **18**, 141. (10.1186/s12879-018-3052-6) 29587649 PMC5872497

[158] Gething PW et al. 2012 A long neglected world malaria map: *Plasmodium vivax* endemicity in 2010. PLoS Negl. Trop. Dis. **6**, e1814. (10.1371/journal.pntd.0001814) 22970336 PMC3435256

[159] Gething PW, Patil AP, Smith DL, Guerra CA, Elyazar IR, Johnston GL, Tatem AJ, Hay SI. 2011 A new world malaria map: *Plasmodium falciparum* endemicity in 2010. Malar. J. **10**, 1-16. (10.1186/1475-2875-10-378) 22185615 PMC3274487

[160] Hay SI et al. 2009 A world malaria map: *Plasmodium falciparum* endemicity in 2007. PLoS Med. **6**, e1000048. (10.1371/journal.pmed.1000048) 19323591 PMC2659708

[161] Alegana VA et al. 2016 Advances in mapping malaria for elimination: fine resolution modelling of *Plasmodium falciparum* incidence. Sci. Rep. **6**, 1-14. (10.1038/s41598-016-0001-8) 27405532 PMC4942778

[162] Fornace KM et al. 2016 Association between landscape factors and spatial patterns of *Plasmodium knowlesi* infections in Sabah, Malaysia. Emerg. Infect. Dis. **22**, 201-208. (10.3201/eid2202.150656) 26812373 PMC4734530

[163] Reid H et al. 2010 Baseline spatial distribution of malaria prior to an elimination programme in Vanuatu. Malar. J. **9**, 150. (10.1186/1475-2875-9-150) 20525209 PMC2893196

[164] Stensgaard AS, Vounatsou P, Onapa AW, Simonsen PE, Pedersen EM, Rahbek C, Kristensen TK. 2011 Bayesian geostatistical modelling of malaria and lymphatic filariasis infections in Uganda: predictors of risk and geographical patterns of co-endemicity. Malar. J. **10**, 298. (10.1186/1475-2875-10-298) 21989409 PMC3216645

[165] Amratia P, Psychas P, Abuaku B, Ahorlu C, Millar J, Oppong S, Koram K, Valle D. 2019 Characterizing local-scale heterogeneity of malaria risk: a case study in Bunkpurugu-Yunyoo district in northern Ghana. Malar. J. **18**, 81. (10.1186/s12936-019-2703-4) 30876413 PMC6420752

[166] Diggle P, Moyeed R, Rowlingson B, Thomson M. 2002 Childhood malaria in the Gambia: a case-study in model-based geostatistics. J. R. Stat. Soc. Ser. C Appl. Stat. **51**, 493-506. (10.1111/1467-9876.00283)

[167] Giorgi E, Sesay SSS, Terlouw DJ, Diggle PJ. 2015 Combining data from multiple spatially referenced prevalence surveys using generalized linear geostatistical models. J. R. Stat. Soc. Ser. C Stat. Soc. **178**, 445-464. (10.1111/rssa.12069)

[168] Craig MH, Sharp BL, Mabaso ML, Kleinschmidt I. 2007 Developing a spatial-statistical model and map of historical malaria prevalence in Botswana using a staged variable selection procedure. Int. J. Health Geogr. **6**, 1-15. (10.1186/1476-072X-6-44) 17892584 PMC2082025

[169] Giardina F, Kasasa S, Sié A, Utzinger J, Tanner M, Vounatsou P. 2014 Effects of vector-control interventions on changes in risk of malaria parasitaemia in sub-Saharan Africa: a spatial and temporal analysis. Lancet Glob. Health **2**, e601-e615. (10.1016/S2214-109X(14)70300-6) 25304636

[170] Ashton RA et al. 2015 Geostatistical modeling of malaria endemicity using serological indicators of exposure collected through school surveys. Am. J. Trop. Med. Hyg. **93**, 168-177. (10.4269/ajtmh.14-0620) 25962770 PMC4497890

[171] Giardina F, Franke J, Vounatsou P. 2015 Geostatistical modelling of the malaria risk in Mozambique: effect of the spatial resolution when using remotely-sensed imagery. Geospat. Health **10**, 232-238. (10.4081/gh.2015.333) 26618310

[172] Aimone AM, Brown P, Owusu-Agyei S, Zlotkin SH, Cole DC. 2017 Impact of iron fortification on the geospatial patterns of malaria and non-malaria infection risk among young children: a secondary spatial analysis of clinical trial data from Ghana. BMJ Open **7**, e013192. (10.1136/bmjopen-2016-013192) PMC5734205 28592572

[173] Noor AM, Alegana VA, Kamwi RN, Hansford CF, Ntomwa B, Katokele S, Snow RW. 2013 Malaria control and the intensity of *Plasmodium falciparum* transmission in Namibia 1969–1992. PLoS ONE 2013/05/15 ed. **8**, e63350. (10.1371/journal.pone.0063350) 23667604 PMC3646760

[174] Reid H, Haque U, Clements AC, Tatem AJ, Vallely A, Ahmed SM, Islam A, Haque R. 2010 Mapping malaria risk in Bangladesh using Bayesian geostatistical models. Am. J. Trop. Med. Hyg. 2010/10/05 ed. **83**, 861-867. (10.4269/ajtmh.2010.10-0154) 20889880 PMC2946757

[175] Nguyen M et al. 2020 Mapping malaria seasonality in Madagascar using health facility data. BMC Med. **18**, 1-11. (10.1186/s12916-019-1443-1) 32036785 PMC7008536

[176] Arab A, Jackson MC, Kongoli C. 2014 Modelling the effects of weather and climate on malaria distributions in West Africa. Malar. J. **13**, 126. (10.1186/1475-2875-13-126) 24678602 PMC3976358

[177] Samadoulougou S, Maheu-Giroux M, Kirakoya-Samadoulougou F, De Keukeleire M, Castro MC, Robert A. 2014 Multilevel and geo-statistical modeling of malaria risk in children of Burkina Faso. Parasit. Vectors **7**, 350. (10.1186/1756-3305-7-350) 25074132 PMC4262087

[178] Elyazar IR, Gething PW, Patil AP, Rogayah H, Kusriastuti R, Wismarini DM, Tarmizi SN, Baird JK, Hay SI. 2011 *Plasmodium falciparum* malaria endemicity in Indonesia in 2010. PLoS ONE 2011/07/09 ed. **6**, e21315. (10.1371/journal.pone.0021315) 21738634 PMC3126795

[179] Gething PW, Patil AP, Hay SI. 2010 Quantifying aggregated uncertainty in *Plasmodium falciparum* malaria prevalence and populations at risk via efficient space-time geostatistical joint simulation. PLoS Comput. Biol. **6**, e1000724. (10.1371/journal.pcbi.1000724) 20369009 PMC2848537

[180] Hanks EM, Schliep EM, Hooten MB, Hoeting JA. 2015 Restricted spatial regression in practice: geostatistical models, confounding, and robustness under model misspecification. Environmetrics **26**, 243-254. (10.1002/env.2331)

[181] Chiaravalloti-Neto F et al. 2019 Seroprevalence for dengue virus in a hyperendemic area and associated socioeconomic and demographic factors using a cross-sectional design and a geostatistical approach, state of Sao Paulo, Brazil. BMC Infect. Dis. **19**, 441. (10.1186/s12879-019-4074-4) 31109295 PMC6528304

[182] Kazembe LN, Kleinschmidt I, Holtz TH, Sharp BL. 2006 Spatial analysis and mapping of malaria risk in Malawi using point-referenced prevalence of infection data. Int. J. Health Geogr. **5**, 41. (10.1186/1476-072X-5-41) 16987415 PMC1584224

[183] Ayele DG, Zewotir TT, Mwambi HG. 2013 Spatial distribution of malaria problem in three regions of Ethiopia. Malar. J. **12**, 207. (10.1186/1475-2875-12-207) 23773317 PMC3703284

[184] Noor AM, Clements AC, Gething PW, Moloney G, Borle M, Shewchuk T, Hay SI, Snow RW. 2008 Spatial prediction of *Plasmodium falciparum* prevalence in Somalia. Malar. J. 2008/08/23 ed. **7**, 159. (10.1186/1475-2875-7-159) 18717998 PMC2531188

[185] Macharia PM, Giorgi E, Noor AM, Waqo E, Kiptui R, Okiro EA, Snow RW. 2018 Spatio-temporal analysis of *Plasmodium falciparum* prevalence to understand the past and chart the future of malaria control in Kenya. Malar. J. **17**, 1-13. (10.1186/s12936-018-2489-9) 30257697 PMC6158896

[186] Kang SY et al. 2018 Spatio-temporal mapping of Madagascar's Malaria Indicator Survey results to assess *Plasmodium falciparum* endemicity trends between 2011 and 2016. BMC Med. **16**, 1-15. (10.1186/s12916-017-0981-7) PMC5964908 29788968

[187] Colborn KL, Giorgi E, Monaghan AJ, Gudo E, Candrinho B, Marrufo TJ, Colborn JM. 2018 Spatio-temporal modelling of weekly malaria incidence in children under 5 for early epidemic detection in Mozambique. Sci. Rep. **8**, 1-9. (10.1038/s41598-018-27537-4) 29915366 PMC6006329

[188] Ssempiira J, Nambuusi B, Kissa J, Agaba B, Makumbi F, Kasasa S, Vounatsou P. 2017 The contribution of malaria control interventions on spatio-temporal changes of parasitaemia risk in Uganda during 2009–2014. Parasit. Vectors **10**, 450. (10.1186/s13071-017-2393-0) 28964263 PMC5622426

[189] Noor AM et al. 2013 The receptive versus current risks of *Plasmodium falciparum* transmission in Northern Namibia: implications for elimination. BMC Infect. Dis. **13**, 1-10. (10.1186/1471-2334-13-1) 23617955 PMC3639180

[190] Matthys B et al. 2006 Urban farming and malaria risk factors in a medium-sized town in Côte d'Ivoire. Am. J. Trop. Med. Hyg. **75**, 1223-1231. (10.4269/ajtmh.2006.75.1223) 17172397

[191] Kleinschmidt I, Sharp BL, Clarke GP, Curtis B, Fraser C. 2001 Use of generalized linear mixed models in the spatial analysis of small-area malaria incidence rates in Kwazulu Natal, South Africa. Am. J. Epidemiol. **153**, 1213-1221. (10.1093/aje/153.12.1213) 11415957

[192] Giorgi E, Osman AA, Hassan AH, Ali AA, Ibrahim F, Amran JG, Noor AM, Snow RW. 2018 Using non-exceedance probabilities of policy-relevant malaria prevalence thresholds to identify areas of low transmission in Somalia. Malar. J. **17**, 1-10. (10.1186/s12936-018-2238-0) 29463264 PMC5819647

[193] Chirombo J, Lowe R, Kazembe L. 2014 Using structured additive regression models to estimate risk factors of malaria: analysis of 2010 Malawi malaria indicator survey data. PLoS ONE **9**, e101116. (10.1371/journal.pone.0101116) 24991915 PMC4084636

[194] Guerra CA et al. 2019 Human mobility patterns and malaria importation on Bioko Island. Nat. Commun. **10**, 1-10. (10.1038/s41467-019-10339-1) 31133635 PMC6536527

[195] Fornace KM et al. 2019 Local human movement patterns and land use impact exposure to zoonotic malaria in Malaysian Borneo. Elife **8**, e47602. (10.7554/eLife.47602) 31638575 PMC6814363

[196] Raso G et al. 2012 Mapping malaria risk among children in Côte d'Ivoire using Bayesian geo-statistical models. Malar. J. **11**, 1-11. (10.1186/1475-2875-11-160) 22571469 PMC3483263

[197] Gosoniu L, Vounatsou P. 2011 Non-stationary partition modeling of geostatistical data for malaria risk mapping. J. Appl. Stat. **38**, 3-13. (10.1080/02664760903008961)

[198] Cohen JM, Dlamini S, Novotny JM, Kandula D, Kunene S, Tatem AJ. 2013 Rapid case-based mapping of seasonal malaria transmission risk for strategic elimination planning in Swaziland. Malar. J. 2013/02/13 ed. **12**, 61. (10.1186/1475-2875-12-61) 23398628 PMC3637471

[199] Valle D, Lima JMT. 2014 Large-scale drivers of malaria and priority areas for prevention and control in the Brazilian Amazon region using a novel multi-pathogen geospatial model. Malar. J. **13**, 13. (10.1186/1475-2875-13-443) 25412882 PMC4247612

[200] Gosoniu L, Vounatsou P, Sogoba N, Smith T. 2006 Bayesian modelling of geostatistical malaria risk data. Geospat. Health 2008/08/08 ed. **1**, 127-139. (10.4081/gh.2006.287) 18686238

[201] Giardina F, Gosoniu L, Konate L, Diouf MB, Perry R, Gaye O, Faye O, Vounatsou P. 2012 Estimating the burden of malaria in Senegal: Bayesian zero-inflated binomial geostatistical modeling of the MIS 2008 Data. PLoS ONE **7**, e32625. (10.1371/journal.pone.0032625) 22403684 PMC3293829

[202] Janko M, Goel V, Emch M. 2019 Extending multilevel spatial models to include spatially varying coefficients. Health Place **60**, 102235. (10.1016/j.healthplace.2019.102235) 31778846 PMC6903407

[203] Riedel N, Vounatsou P, Miller JM, Gosoniu L, Chizema-Kawesha E, Mukonka V, Steketee RW. 2010 Geographical patterns and predictors of malaria risk in Zambia: Bayesian geostatistical modelling of the 2006 Zambia national malaria indicator survey (ZMIS). Malar. J. **9**, 37. (10.1186/1475-2875-9-37) 20122148 PMC2845589

[204] Gosoniu L, Vounatsou P, Sogoba N, Maire N, Smith T. 2009 Mapping malaria risk in West Africa using a Bayesian nonparametric non-stationary model. Comput. Stat. Data Anal. **53**, 3358-3371. (10.1016/j.csda.2009.02.022)

[205] Salehi M, Mohammad K, Farahani MM, Zeraati H, Nourijelyani K, Zayeri F. 2008 Spatial modeling of malaria incidence rates in Sistan and Baluchistan province, Islamic Republic of Iran. Saudi Med. J. **29**, 1791-1796. 19082235

[206] Raso G, Silué KD, Vounatsou P, Singer BH, Yapi A, Tanner M, Utzinger J, N'Goran EK. 2009 Spatial risk profiling of *Plasmodium falciparum* parasitaemia in a high endemicity area in Côte d'Ivoire. Malar. J. **8**, 252. (10.1186/1475-2875-8-252) 19906295 PMC2783037

[207] Adegboye OA, Leung DHY, Wang YG. 2017 Analysis of spatial data with a nested correlation structure. J. R. Stat. Soc. Ser. C Appl. Stat. **67**, 329-354. (10.1111/rssc.12230)

[208] Sharmin S, Glass K, Viennet E, Harley D. 2018 Geostatistical mapping of the seasonal spread of under-reported dengue cases in Bangladesh. PLoS Negl. Trop. Dis. **12**, e0006947. (10.1371/journal.pntd.0006947) 30439942 PMC6264868

[209] Sow A et al. 2020 Changes in the transmission dynamic of chikungunya virus in Southeastern Senegal. Viruses **12**, 196. (10.3390/v12020196) PMC7077306 32050663

[210] Ahmad H, Ali A, Fatima SH, Zaidi F, Khisroon M, Rasheed SB, Ullah I, Ullah S, Shakir M. 2020 Spatial modeling of dengue prevalence and kriging prediction of dengue outbreak in Khyber Pakhtunkhwa (Pakistan) using presence only data. Stoch. Environ. Res. Risk Assess. **34**, 1023-1036. (10.1007/s00477-020-01818-9)

[211] Routledge I et al. 2020 Tracking progress towards malaria elimination in China: individual-level estimates of transmission and its spatiotemporal variation using a diffusion network approach. PLoS Comput. Biol. **16**, e1007707. (10.1371/journal.pcbi.1007707) 32203520 PMC7117777

[212] Sedda L, Taylor BM, Eiras AE, Marques JT, Dillon RJ. 2020 Using the intrinsic growth rate of the mosquito population improves spatio-temporal dengue risk estimation. Acta Trop. **208**, 105519. (10.1016/j.actatropica.2020.105519) 32389450 PMC7315132

[213] Rue H, Held L. 2005 Gaussian Markov random fields: theory and applications. Boca Raton, FL: CRC press.

[214] Matérn B. 2013 Spatial variation, vol. 36. New York, NY: Springer Science & Business Media.

[215] Chiaravalloti-Neto F, Pereira M, Fávaro EA, Dibo MR, Mondini A, Rodrigues-Junior AL, Chierotti AP, Nogueira M. 2015 Assessment of the relationship between entomologic indicators of *Aedes aegypti* and the epidemic occurrence of dengue virus 3 in a susceptible population, São José do Rio Preto, São Paulo, Brazil. Acta Trop. **142**, 167-177. (10.1016/j.actatropica.2014.11.017) 25484110

[216] Farinelli EC, Baquero OS, Stephan C, Chiaravalloti-Neto F. 2018 Low socioeconomic condition and the risk of dengue fever: a direct relationship. Acta Trop. **180**, 47-57. (10.1016/j.actatropica.2018.01.005) 29352990

[217] Charlwood JD, Tomas EVE, Braganca M, Cuamba N, Alifrangis M, Stanton M. 2015 Malaria prevalence and incidence in an isolated, meso-endemic area of Mozambique. Peerj **3**, 22. (10.7717/peerj.1370) PMC4647567 26587341

[218] Ugwu CLJ, Zewotir T. 2020 Evaluating the effects of climate and environmental factors on under-5 children malaria spatial distribution using generalized additive models (GAMs). J. Epidemiol. Glob. Health **10**, 304-314. (10.2991/jegh.k.200814.001) 33009733 PMC7758859

[219] Mutucumarana CP et al. 2020 Geospatial analysis of dengue emergence in rural areas in the Southern Province of Sri Lanka. Trans. R Soc. Trop. Med. Hyg. **114**, 408-414. (10.1093/trstmh/trz123) 31885050 PMC7528758

[220] Vazquez-Prokopec GM, Kitron U, Montgomery B, Horne P, Ritchie SA. 2010 Quantifying the spatial dimension of dengue virus epidemic spread within a tropical urban environment. PLoS Negl. Trop. Dis. **4**, e920. (10.1371/journal.pntd.0000920) 21200419 PMC3006131

[221] Braga C, Luna CF, Martelli CM, de Souza WV, Cordeiro MT, Alexander N, Júnior JC, Marques ET. 2010 Seroprevalence and risk factors for dengue infection in socio-economically distinct areas of Recife, Brazil. Acta Trop. **113**, 234-240. (10.1016/j.actatropica.2009.10.021) 19896921 PMC3847853

[222] Honório NA et al. 2009 Spatial evaluation and modeling of dengue seroprevalence and vector density in Rio de Janeiro, Brazil. PLoS Negl. Trop. Dis. **3**, e545. (10.1371/journal.pntd.0000545) 19901983 PMC2768822

[223] Siqueira-Junior JB, Maciel IJ, Barcellos C, Souza WV, Carvalho MS, Nascimento NE, Oliveira RM, Morais-Neto O, Martelli CMT. 2008 Spatial point analysis based on dengue surveys at household level in central Brazil. BMC Public Health **8**, 361. (10.1186/1471-2458-8-361) 18937868 PMC2576465

[224] Chien LC, Sy F, Pérez A. 2019 Identifying high risk areas of Zika virus infection by meteorological factors in Colombia. BMC Infect. Dis. **19**, 888. (10.1186/s12879-019-4499-9) 31651247 PMC6814059

[225] Hundessa S et al. 2018 Projecting potential spatial and temporal changes in the distribution of *Plasmodium vivax* and *Plasmodium falciparum* malaria in China with climate change. Sci. Total Environ. **627**, 1285-1293. (10.1016/j.scitotenv.2018.01.300) 30283159 PMC6166864

[226] Kazembe LN, Mathanga DP. 2016 Estimating risk factors of urban malaria in Blantyre, Malawi: a spatial regression analysis. Asian Pac. J. Trop. Biomed. **6**, 376-381. (10.1016/j.apjtb.2016.03.011)

[227] Cissoko M et al. 2020 Geo-epidemiology of malaria at the health area level, dire health district, Mali, 2013–2017. Int. J. Environ. Res. Public Health **17**, 3982. (10.3390/ijerph17113982) PMC7312793 32512740

[228] Wood SN. 2017 Generalized additive models: an introduction with R. Boca Raton, FL: CRC Press.

[229] Watts MJ, Kotsila P, Mortyn PG, Sarto i Monteys V, Urzi Brancati C. 2020 Influence of socio-economic, demographic and climate factors on the regional distribution of dengue in the United States and Mexico. Int. J. Health Geogr. **19**, 44. (10.1186/s12942-020-00241-1) 33138827 PMC7607660

[230] Laguna F, Grillet ME, León JR, Ludeña C. 2017 Modelling malaria incidence by an autoregressive distributed lag model with spatial component. Spat. Spatio-Temporal Epidemiol. **22**, 27-37. (10.1016/j.sste.2017.05.001) 28760265

[231] Rue H, Martino S, Chopin N. 2009 Approximate Bayesian inference for latent Gaussian models by using integrated nested Laplace approximations. J. R. Stat. Soc. Ser. B Stat. Methodol. **71**, 319-92. (10.1111/j.1467-9868.2008.00700.x)

[232] Lunn DJ, Thomas A, Best N, Spiegelhalter D. 2000 WinBUGS—a Bayesian modelling framework: concepts, structure, and extensibility. Stat. Comput. **10**, 325-337. (10.1023/A:1008929526011)

[233] Halim S, Handojo A, Widodo I, Octavia T. 2020 Spatial multi-layer perceptron model for predicting dengue fever outbreaks in Surabaya. Adv. Sci. Technol. Eng. Syst. J. **5**, 103-108. (10.25046/aj050514)

[234] Akhtar M, Kraemer MUG, Gardner LM. 2019 A dynamic neural network model for predicting risk of Zika in real time. BMC Med. **17**, 171. (10.1186/s12916-019-1389-3) 31474220 PMC6717993

[235] Haddawy P, Hasan AHMI, Kasantikul R, Lawpoolsri S, Sa-angchai P, Kaewkungwal J, Singhasivanon P. 2018 Spatiotemporal Bayesian networks for malaria prediction. Artif. Intell. Med. **84**, 127-138. (10.1016/j.artmed.2017.12.002) 29241658

[236] Rossi G, Karki S, Smith RL, Brown WM, Ruiz MO. 2018 The spread of mosquito-borne viruses in modern times: a spatio-temporal analysis of dengue and chikungunya. Spat. Spatio-Temporal Epidemiol. **26**, 113-125. (10.1016/j.sste.2018.06.002) 30390927

[237] Shi B, Liu J, Zhou X-N, Yang G-J. 2014 Inferring plasmodium vivax transmission networks from tempo-spatial surveillance data. PLoS Negl. Trop. Dis. **8**, e2682. (10.1371/journal.pntd.0002682) 24516684 PMC3916251

[238] Simini F, González MC, Maritan A, Barabási A-L. 2012 A universal model for mobility and migration patterns. Nature **484**, 96-100. (10.1038/nature10856) 22367540

[239] Zhang Q et al. 2017 Spread of Zika virus in the Americas. Proc. Natl Acad. Sci. USA **114**, E4334-E4343. (10.1073/pnas.1620161114) 28442561 PMC5465916

[240] Pizzitutti F, Pan W, Barbieri A, Miranda JJ, Feingold B, Guedes GR, Alarcon-Valenzuela J, Mena CF. 2015 A validated agent-based model to study the spatial and temporal heterogeneities of malaria incidence in the rainforest environment. Malar. J. **14**, 1030. (10.1186/s12936-015-1030-7) PMC4688926 26696294

[241] Zhu G et al. 2019 Effects of human mobility, temperature and mosquito control on the spatiotemporal transmission of dengue. Sci. Total Environ. **651**, 969-978. (10.1016/j.scitotenv.2018.09.182) 30360290

[242] Silal SP, Little F, Barnes KI, White LJ. 2015 Hitting a moving target: a model for malaria elimination in the presence of population movement. PLoS ONE **10**, e0144990. (10.1371/journal.pone.0144990) 26689547 PMC4686217

[243] Senapati A, Sardar T, Ganguly KS, Ganguly KS, Chattopadhyay AK, Chattopadhyay J. 2019 Impact of adult mosquito control on dengue prevalence in a multi-patch setting: a case study in Kolkata (2014–2015). J. Theor. Biol. **478**, 139-152. (10.1016/j.jtbi.2019.06.021) 31229456

[244] Xue L, Scott HM, Cohnstaedt LW, Scoglio C. 2012 A network-based meta-population approach to model Rift Valley fever epidemics. J. Theor. Biol. **306**, 129-144. (10.1016/j.jtbi.2012.04.029) 22564391

[245] Karl S, Halder N, Kelso JK, Ritchie SA, Milne GJ. 2014 A spatial simulation model for dengue virus infection in urban areas. BMC Infect. Dis. **14**, 1-17. (10.1186/1471-2334-14-447) 25139524 PMC4152583

[246] Moulay D, Pigné Y. 2013 A metapopulation model for chikungunya including populations mobility on a large-scale network. J. Theor. Biol. **318**, 129-139. (10.1016/j.jtbi.2012.11.008) 23154189

[247] Massaro E, Kondor D, Ratti C. 2019 Assessing the interplay between human mobility and mosquito borne diseases in urban environments. Sci. Rep. **9**, 1-13. (10.1038/s41598-019-53127-z) 31729435 PMC6858332

[248] Wesolowski A, Qureshi T, Boni MF, Sundsøy PR, Johansson MA, Rasheed SB, Engø-Monsen K, Buckee CO. 2015 Impact of human mobility on the emergence of dengue epidemics in Pakistan. Proc. Natl Acad. Sci. **112**, 11 887-11 892. (10.1073/pnas.1504964112) PMC4586847 26351662

[249] Sun K et al. 2018 Quantifying the risk of local Zika virus transmission in the contiguous US during the 2015–2016 ZIKV epidemic. BMC Med. **16**, 195. (10.1186/s12916-018-1185-5) 30336778 PMC6194624

[250] Gardner LM, Bota A, Gangavarapu K, Kraemer MUG, Grubaugh ND. 2018 Inferring the risk factors behind the geographical spread and transmission of Zika in the Americas. PLoS Negl. Trop. Dis. **12**, 25. (10.1371/journal.pntd.0006194) PMC5790294 29346387

[251] Seroussi I, Levy N, Yom-Tov E. 2020 Multi-season analysis reveals the spatial structure of disease spread. Phys. Stat. Mech. Appl. **547**, 124425. (10.1016/j.physa.2020.124425)

[252] Barrios E, Lee S, Vasilieva O. 2018 Assessing the effects of daily commuting in two-patch dengue dynamics: a case study of Cali, Colombia. J. Theor. Biol. **453**, 14-39. (10.1016/j.jtbi.2018.05.015) 29775680

[253] Stolerman LM, Coombs D, Boatto S. 2015 SIR-network model and its application to dengue fever. SIAM J. Appl. Math. **75**, 2581-2609. (10.1137/140996148)

[254] Zhu G et al. 2018 The spatiotemporal transmission of dengue and its driving mechanism: a case study on the 2014 dengue outbreak in Guangdong, China. Sci. Total Environ. 622–623, 252-259. (10.1016/j.scitotenv.2017.11.314) 29216466

[255] Chadsuthi S, Althouse BM, Iamsirithaworn S, Triampo W, Grantz KH, Cummings DAT. 2018 Travel distance and human movement predict paths of emergence and spatial spread of chikungunya in Thailand. Epidemiol. Infect. **146**, 1654-1662. (10.1017/S0950268818001917) 29983134 PMC9507951

[256] Kim M, Paini D, Jurdak R. 2019 Modeling stochastic processes in disease spread across a heterogeneous social system. Proc. Natl Acad. Sci. USA **116**, 401-406. (10.1073/pnas.1801429116) 30587583 PMC6329989

[257] Zhu G, Liu J, Tan Q, Shi B. 2016 Inferring the spatio-temporal patterns of dengue transmission from surveillance data in Guangzhou, China. PLoS Negl. Trop. Dis. **10**, e0004633. (10.1371/journal.pntd.0004633) 27105350 PMC4841561

[258] Li R, Xu L, Bjørnstad ON, Liu K, Song T, Chen A, Xu B, Liu Q, Stenseth NC. 2019 Climate-driven variation in mosquito density predicts the spatiotemporal dynamics of dengue. Proc. Natl Acad. Sci. USA **116**, 3624-3629. (10.1073/pnas.1806094116) 30808752 PMC6397594

[259] Marini G, Guzzetta G, Marques Toledo CA, Teixeira M, Rosa R, Merler S. 2019 Effectiveness of ultra-low volume insecticide spraying to prevent dengue in a non-endemic metropolitan area of Brazil. PLoS Comput. Biol. **15**, e1006831. (10.1371/journal.pcbi.1006831) 30849074 PMC6426269

[260] Guzzetta G, Marques-Toledo CA, Rosà R, Teixeira M, Merler S. 2018 Quantifying the spatial spread of dengue in a non-endemic Brazilian metropolis via transmission chain reconstruction. Nat. Commun. **9**, 2837. (10.1038/s41467-018-05230-4) 30026544 PMC6053439

[261] Yu HL, Angulo JM, Cheng MH, Wu J, Christakos G. 2014 An online spatiotemporal prediction model for dengue fever epidemic in Kaohsiung (Taiwan). Biom. J. **56**, 428-440. (10.1002/bimj.201200270) 24615833

[262] O'Reilly KM et al. 2018 Projecting the end of the Zika virus epidemic in Latin America: a modelling analysis. BMC Med. **16**, 180. (10.1186/s12916-018-1158-8) 30285863 PMC6169075

[263] Ruktanonchai NW, DeLeenheer P, Tatem AJ, Alegana VA, Caughlin TT, Erbach-Schoenberg Ez, Lourenço C, Ruktanonchai CW, Smith DL. 2016 Identifying malaria transmission foci for elimination using human mobility data. PLoS Comput. Biol. **12**, e1004846. (10.1371/journal.pcbi.1004846) 27043913 PMC4820264

[264] Fitzgibbon WE, Morgan JJ, Webb GF. 2017 An outbreak vector–host epidemic model with spatial structure: the 2015–2016 Zika outbreak in Rio De Janeiro. Theor. Biol. Med. Model. **14**, 7. (10.1186/s12976-017-0051-z) 28347332 PMC5368945

[265] Miyaoka TY, Lenhart S, Meyer JFCA. 2019 Optimal control of vaccination in a vector-borne reaction–diffusion model applied to Zika virus. J. Math. Biol. **79**, 1077-1104. (10.1007/s00285-019-01390-z) 31187254

[266] Mukhtar AYA, Munyakazi JB, Ouifki R. 2020 Assessing the role of human mobility on malaria transmission. Math. Biosci. **320**, 108304. (10.1016/j.mbs.2019.108304) 31883985

[267] Gerardin J, Bever CA, Hamainza B, Miller JM, Eckhoff PA, Wenger EA. 2016 Optimal population-level infection detection strategies for malaria control and elimination in a spatial model of malaria transmission. PLoS Comput. Biol. **12**, e1004707. (10.1371/journal.pcbi.1004707) 26764905 PMC4713231

[268] Núñez-López M, Alarcón Ramos L, Velasco-Hernández JX. 2021 Migration rate estimation in an epidemic network. Appl. Math. Model **89**, 1949-1964. (10.1016/j.apm.2020.08.025) 32952269 PMC7486824

[269] Moore SM et al. 2018 Local and regional dynamics of chikungunya virus transmission in Colombia: the role of mismatched spatial heterogeneity. BMC Med. **16**, 1-6. (10.1186/s12916-018-1127-2) PMC6116375 30157921

[270] Jordan MI, Mitchell TM. 2015 Machine learning: trends, perspectives, and prospects. Science **349**, 255-260. (10.1126/science.aaa8415) 26185243

[271] Baker RE, Peña J-M, Jayamohan J, Jérusalem A. 2018 Mechanistic models versus machine learning, a fight worth fighting for the biological community? Biol. Lett. **14**, 20170660. (10.1098/rsbl.2017.0660) 29769297 PMC6012710

[272] Stoddard ST, Morrison AC, Vazquez-Prokopec GM, Soldan VP, Kochel TJ, Kitron U, Elder JP, Scott TW. 2009 The role of human movement in the transmission of vector-borne pathogens. PLoS Negl. Trop. Dis. **3**, e481. (10.1371/journal.pntd.0000481) 19621090 PMC2710008

[273] Besag J, York J, Mollié A. 1991 Bayesian image restoration, with two applications in spatial statistics. Ann. Inst. Stat. Math. **43**, 1-20. (10.1007/BF00116466)

[274] Stoddard ST et al. 2013 House-to-house human movement drives dengue virus transmission. Proc. Natl Acad. Sci. USA **110**, 994-999. (10.1073/pnas.1213349110) 23277539 PMC3549073

[275] Dambach P, Schleicher M, Korir P, Ouedraogo S, Dambach J, Sié A, Dambach M, Becker N. 2018 Nightly biting cycles of *Anopheles* species in rural northwestern Burkina Faso. J. Med. Entomol. **55**, 1027-1034. (10.1093/jme/tjy043) 29635478 PMC6025195

[276] Kabbale FG, Akol AM, Kaddu JB, Onapa AW. 2013 Biting patterns and seasonality of *Anopheles gambiae* *sensu lato* and *Anopheles funestus* mosquitoes in Kamuli District, Uganda. Parasit. Vectors **6**, 340. (10.1186/1756-3305-6-340) 24304974 PMC3866981

[277] Pindolia DK, Garcia AJ, Wesolowski A, Smith DL, Buckee CO, Noor AM, Snow RW, Tatem AJ. 2012 Human movement data for malaria control and elimination strategic planning. Malar. J. **11**, 205. (10.1186/1475-2875-11-205) 22703541 PMC3464668

[278] Kraemer MUG, Perkins TA, Cummings DA, Zakar R, Hay SI, Smith DL, Reiner Jr RC. 2015 Big city, small world: density, contact rates, and transmission of dengue across Pakistan. J. R Soc. Interface **12**, 20150468. (10.1098/rsif.2015.0468) 26468065 PMC4614486

[279] Tizzoni M, Bajardi P, Decuyper A, Kon Kam King G, Schneider CM, Blondel V, Smoreda Z, González MC, Colizza V. 2014 On the use of human mobility proxies for modeling epidemics. PLoS Comput. Biol. **10**, e1003716. (10.1371/journal.pcbi.1003716) 25010676 PMC4091706

[280] Lee SA et al. 2021 Data and R code to accompany ‘Spatial connectivity in mosquito-borne disease models: a systematic review of methods and assumptions’ (version v1.0.0). (Version V1.0.0). Zenodo. (10.5281/zenodo.4706866)

